# Activation of the Cell Wall Integrity Pathway Promotes Escape from G2 in the Fungus *Ustilago maydis*

**DOI:** 10.1371/journal.pgen.1001009

**Published:** 2010-07-01

**Authors:** Natalia Carbó, José Pérez-Martín

**Affiliations:** Department of Microbial Biotechnology, National Center of Biotechnology, Consejo Superior de Investigaciones Científicas, Madrid, Spain; National Institute of Diabetes and Digestive and Kidney Diseases, United States of America

## Abstract

It is widely accepted that MAPK activation in budding and fission yeasts is often associated with negative effects on cell cycle progression, resulting in delay or arrest at a specific stage in the cell cycle, thereby enabling cells to adapt to changing environmental conditions. For instance, activation of the Cell Wall Integrity (CWI) pathway in the budding yeast *Saccharomyces cerevisiae* signals an increase in CDK inhibitory phosphorylation, which leads cells to remain in the G2 phase. Here we characterized the CWI pathway of *Ustilago maydis*, a fungus evolutionarily distant from budding and fission yeasts, and show that activation of the CWI pathway forces cells to escape from G2 phase. In spite of these disparate cell cycle responses in *S. cerevisiae* and *U. maydis*, the CWI pathway in both organisms appears to respond to the same class cell wall stressors. To understand the basis of such a difference, we studied the mechanism behind the *U. maydis* response. We found that activation of CWI pathway in *U. maydis* results in a decrease in CDK inhibitory phosphorylation, which depends on the mitotic phosphatase Cdc25. Moreover, in response to activation of the CWI pathway, Cdc25 accumulates in the nucleus, providing a likely explanation for the increase in the unphosphorylated form of CDK. We also found that the extended N-terminal domain of Cdc25, which is dispensable under normal growth conditions, is required for this G2 escape as well as for resistance to cell wall stressors. We propose that the process of cell cycle adaptation to cell stress evolved differently in these two divergent organisms so that each can move towards a cell cycle phase most appropriate for responding to the environmental signals encountered.

## Author Summary

Signaling cascades connect changes in the extracellular environment with cellular responses. An obvious target for this signaling is cell cycle machinery, since cells have to integrate outside signals to decide whether to proliferate or not. Here we report that the response to cell wall damage in the fungus *Ustilago maydis* forces cells to escape from G2 phase. This finding contrasts with the response observed in *Saccharomyces cerevisiae*, in which cells are kept in G2 phase upon activation of the pathway responding to cell wall damage. We correlated this disparate response to differences in the growth pattern during cell cycle between both fungi, since during apical growth cells are more vulnerable to cell wall instability. While in *S. cerevisiae* polar growth is abrogated during G2 phase, in *U. maydis* pronounced polar growth occurs in G2 phase. Our results suggest that in response to stress stimuli (i. e. cell wall damage), cells move towards the cell cycle stage at which the cellular environment is most appropriate to respond to such stress (i. e. a phase where polar growth is minimal or absent). Depending on the individual organism, this cell cycle stage can differ, thus accounting for the observed differences in outcome.

## Introduction

The mitogen-activated protein kinase (MAPK) family of kinases connects extracellular stimuli with diverse cellular responses [1]. Since progression through the cell cycle is critically dependent on the integration of external signals such as the presence of environmental growth factors or stress stimuli, it seems obvious that cell cycle regulation would be a prime target of MAPK-mediated signaling. In fact, patterns have begun to emerge from a large number of studies showing functions of MAPK subfamilies at different stages of the cell cycle [2]. However, in spite of these studies, a coherent role of MAPK-mediated signaling into cell cycle control has yet to be clarified. MAPK signaling produces both negative and positive effects in cell cycle regulation that depends not only on the stimulus or MAPK pathway implied in the signal transmission, but also on the extent of the MAPK activation and the cell type [3].

Studies carried out in fungal cells also support a role for MAPK pathways in regulating cell cycle [4], [5]. In *Saccharomyces cerevisiae*, which utilizes five separate MAPK cascades to respond appropriately to environmental changes [6], activation of MAPK pathway is often linked to a cell cycle arrest or delay. Activation of Fus3 MAPK cascade (in response to mating pheromone) imposes cell cycle arrest in the G1 phase via phosphorylation of Far1, a cyclin-dependent kinase inhibitor (CKI), which acts on Cdc28, the yeast Cdk1 [7]–[9]. The Hog1 MAPK pathway, in response to hyperosmotic stress also imposes cell cycle arrest both in the G1 and G2 phases via activation of the Sic1 CKI, or blocking the action of a protein kinase (Hsl1) that inhibits the protein kinase Swe1 (the *Schizosaccharomyces pombe* Wee1 ortholog that acts as a mitotic inhibitor), respectively [10], [11]. The Slt2/Mpk1 kinase, which is activated in response to cell wall stress, imposes G2 cell cycle arrest via inhibition (direct or indirect) of the yeast homologue of the Cdc25 phosphatase (Mih1), which is necessary to reverse the inhibitory tyrosine-specific phosphorylation conferred on Cdc28 by Swe1 [12]. Collectively, these observations lead to the general view that in fungi, MAPK activation negatively regulates cell cycle progression, by imposing delays or arrests at specific cell cycle stages to enable the cell to adapt to unfavorable stress conditions or to synchronize cell cycle progression before mating.

Our laboratory has been involved in the characterization of the connections between cell cycle and the induction of the virulence program in the phytopathogenic fungus *Ustilago maydis* [13]. This fungus has been used as a model system in fungal development for many years [14]. The virulence program in *U. maydis* is induced by a pheromone-dependent MAPK cascade [15]–[19]. We reported that activation of this cascade in *U. maydis* also resulted in cell cycle arrest, but in contrast to the well-known G1 cell cycle arrest described in *S. cerevisae* or *S. pombe* [7], [8], [20], [21], pheromone-induced cell cycle arrest in *U. maydis* takes place at the G2 phase [22]. These differences in the regulation of cell cycle arrest may reflect specific features of pheromone response in *U. maydis* or differences in the wiring between signal transduction and cell response in these organisms [23]. Therefore, we looked for cell cycle responses dependent on other MAPK cascades in *U. maydis*. We chose to characterize the Cell Wall Integrity (CWI) pathway, which utilizes MAPK cascades to facilitate the maintenance of the cell wall by mediating cell wall biosynthesis, actin organization and other events necessary to maintain cell wall integrity [24]. As fungal cell growth involves cell wall remodeling that must be coordinated with cell cycle, a connection between cell cycle and CWI pathway would be predicted. Furthermore, as this pathway is conserved among fungi including budding yeast, fission yeast and filamentous fungi [25], such a relationship between CWI pathway and cell cycle control could be of broad interest in fungal physiology.

In this work we report the surprising finding that activation of Mpk1, the MAPK central to CWI cascade, forces cells to escape from G2 phase in *U. maydis*, in contrast to the G2 cell cycle arrest that was reported for *S. cerevisiae* [12]. In both organisms it seems that the Cdc25 phosphatase is the target responsible of this disparate effect.

## Results

### Characterization of the central module of CWI pathway in *U. maydis*

**Figure 1:**
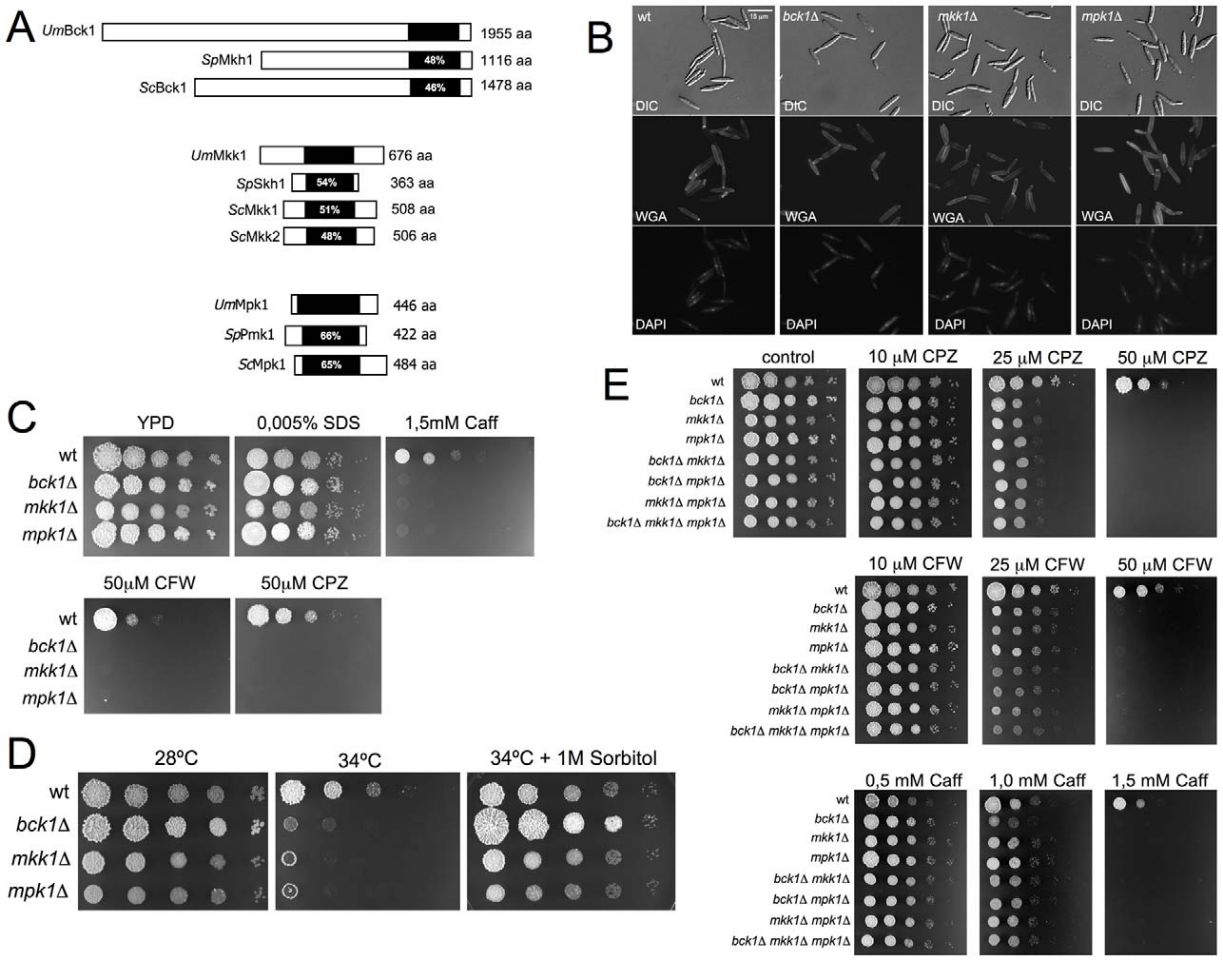
*Ustilago maydis* cell wall integrity pathway. (A) Scheme of the *U. maydis* MEKK, MEK and MAPK proteins in relation to homologous proteins from *Saccharomyces cerevisiae* and *Schizosaccharomyces pombe*. The catalytic domains are shown in black and were identified using the Simple Modular Architecture Research Tool (http://smart.embl-heidelberg.de). The percentages inside each box represent the sequence identity in this domain when compared to the *U. maydis* sequence. (B) Morphology of wild-type (FB1) and *bck1*Δ, *mkk1*Δ and *mpk1*Δ cells growing in nutrient-rich medium, YPD. Cells were stained with 4',6-diamidino-2-phenylindole (DAPI) to visualize the nuclei and FITC-labeled wheat germ agglutinin (WGA), to detect cell wall. Bar 15 µm. (C) Sensitivity of CWI pathway mutant strains to cell wall inhibitors. Cultures of wild-type (FB1) and the indicated strains were grown overnight in rich medium, then diluted to an OD_600_ of 0.5. Tenfold serial dilutions were made and 2 µl of each was plated in solid YPD medium amended with the indicated compounds: Sodium dodecyl sulphate (SDS), caffeine (Caff), Calcofluor White (CFW) and Chlorpromazine (CPZ). The plates were grown for 4 days at 28°C. (D) Growth of FB1 (wt) and CWI pathway mutant strains at 28 and 34°C in solid medium (YPD). At 28°C the growth of control and mutant cells was similar. However, mutant cells showed severe growth defects at 34°C. These defects were alleviated when 1 M Sorbitol was added to culture media. (E) Sensitivity of CWI pathway single, double and triple mutant strains to cell wall inhibitors. Note that no enhancement in sensitivity was obtained when the individual mutations were combined.

We were interested in characterizing the central core of kinases composing the Cell Wall Integrity (CWI) pathway of *U. maydis*. As putative proteins with sequence similarity to MEKK, MEK and MAPK were already annotated in *U. maydis* genome database [26], we performed a phylogenetic analysis to identify the closest homologues to CWI pathway components in *U. maydis* using the CWI core components from *S. cerevisiae* and *S. pombe* for comparison (Figure S1). From this analysis we identified putative homologues to *S. cerevisiae* MEKK (um01662), MEK (um10855), and MAPK (um10107) that were named Bck1, Mkk1 and Mpk1 respectively (Figure 1A).

To address whether these kinase homologues were the canonical components of CWI pathway in *U. maydis*, we generated complete deletions of the corresponding ORFs by PCR-based gene targeting [27]. Mutant strains in each gene as well as control wild-type cells were grown in nutrient rich-medium (YPD) and their duplication time was determined. Also, cells were stained with 4',6-diamidino-2-phenylindole (DAPI) to visualize the nuclei and FITC-labeled wheat germ agglutinin (WGA), a lectin that binds to chitin [28], to detect cell wall. We found no differences between mutant and wild-type cells with respect to doubling time (around 120 min in all cases, not shown) or morphology (Figure 1B). We tested for sensitivity to compounds that affect growth of strains with altered cell wall integrity. Cell dilutions of control and mutant cells were plated on YPD agar media containing different cell envelope stressors (Figure 1C): sodium dodecyl sulphate (SDS), which disrupts the plasma membrane and lyses cells with membrane defects; caffeine (Caff), which has been used extensively to probe signal transduction and cell integrity phenotypes in *S. cerevisiae*; calcofluor white (CFW), which binds to chitin and to a lesser extent glucan and disrupts the cell wall; and chlorpromazine (CPZ), an amphipathic molecule (and potent activator of the CWI pathway in *S. cerevisiae*) that inserts asymmetrically into the plasma membrane causing stretching [24], [29]–[31]. We observed that the *U. maydis* CWI mutant strains were more sensitive to all of these stressors except SDS. We also tested whether the mutant strains were more sensitive to growth at high temperature, as the CWI pathway is essential for the response to high temperature [30], and found this to be the case (Figure 1D). Moreover, we found that the temperature-sensitive growth of the mutant was rescued by 1 M sorbitol, an osmotic stabilizer that can rescue host defects in cell wall integrity [32].

We also constructed double mutant cells carrying different combinations of mutated alleles (*bck1*Δ, *mkk1*Δ; *bck1*Δ, *mpk1*Δ; and *mkk1*Δ, *mpk1*Δ) as well as the triple mutant (*bck1*Δ, *mkk1*Δ, *mpk1*Δ) and tested for enhanced sensitivity to cell wall stressors. The distinct double and triple mutant strains displayed sensitivities that were comparable to single mutants (Figure 1E) consistent with the notion that all these proteins function in a common pathway.

### *U. maydis* Mpk1 is activated after Bck1-Mkk1–mediated phosphorylation

**Figure 2:**
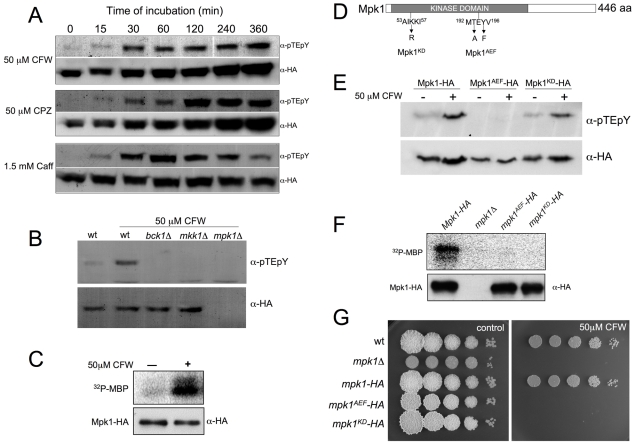
Phosphorylation of Mpk1 T-loop is required for *in vivo* activity. (A) Time course of Mpk1 phosphorylation in response to Calcofluor White (CFW), Chlorpromazine (CPZ) and Caffeine (Caff). Mid log phase cells of a strain carrying a HA-tagged Mpk1 allele (UMA44) were treated with the indicated amount of cell wall stressors and aliquots of the same culture were withdrawn before (0) and at the indicated times (in minutes) following compound addition. Protein extracts were prepared and a similar amount of total protein of each time point were loaded in SDS-PAGE gels and submitted to Western blot assays. The same blot, after stripping, was incubated with anti-pTEpY to detect T-loop phosphorylation and with anti-HA to detect HA-tagged proteins. (B) T-loop phosphorylation of Mpk1 *in vivo* is dependent on upstream kinases. Cell extracts from CFW-treated cultures over a 2 h period were prepared from UMA44 (wt control), UMA44.2 (*bck1*Δ), UMA44.3 (*mkk1*Δ), and UMA3 (*mpk1*Δ), cells and analyzed as described above. (C) Catalytic activity of Mpk1 is enhanced after CFW treatment. HA-tagged proteins were immunoprecipitated from cells extracts prepared from UMA44 cells grown in the presence or absence of 50 µM CFW for 2 h. Protein kinase activity was measured by incubation of immunoprecipitates with purified Myelin Basic Protein (MBP) as substrate and [γ-^32^P] ATP. On the bottom, 8% SDS-PAGE and immunoblot with anti-HA was used to show comparable levels of Mpk1 proteins in the reaction mixtures. On the top, 12.5% SDS-PAGE and autorradiography was used to detect *in vitro* phosphorylated MBP. (D) Scheme of Mpk1 showing the mutated residues in Mpk1^AEF^ and Mpk1^KD^ alleles. (E) T-loop phosphorylation of Mpk1 mutants. Cell extracts from CFW-treated cultures over 2 h period were prepared from UMA44 (Mpk1-HA), UMA59 (Mpk1^AEF^-HA), and UMA60 (Mpk1^KD^-HA), cells and analyzed as described above. (F) Kinase activity of Mpk1 mutants. Immunoprecipitates from UMA44 (*mpk1-HA*), UMA3 (*mpk1*Δ), UMA59 (*mpk1^AEF^-HA*), and UMA60 (*mpk1^KD^-HA*) cells were analyzed as described above. (G) Sensitivity to CFW of Mpk1 mutants.

MAPKs are activated by phosphorylation at a pair of conserved Thr and Tyr residues, located in the so-called T-loop [33], [34]. To correlate the function and regulation of CWI pathway in *U. maydis* with the response to agents that stress the cell wall, we used a commercial specific antibody raised against dually phosphorylated (Thr202/Tyr204)-p44/42 MAPK to monitor the phosphorylation of Thr193 and Tyr195 of *U. maydis* Mpk1. This antibody has been successfully utilized to characterize CWI pathway activation in other fungi [35]–[38]. Previously, we constructed an N-terminal HA tagged *mpk1* allele under the control of the native *mpk1* promoter (*mpk1-HA* allele), and replaced the endogenous copy with this allele. When challenged with the different cell wall stressors the resulting strain was indistinguishable from wild-type cells with respect to sensitivity to these compounds (not shown, see also Figure 2G). We incubated cultures of the strain carrying the HA-tagged Mpk1 allele either with CFW, CPZ or Caff and analyzed the amount of phosphorylated signal at different times by immunoblotting. We observed that the signal corresponding to the phosphorylated form of Mpk1 increased upon the addition of the different cell wall stressors to culture medium (Figure 2A). As it has been reported in other organisms [35]–[37], we found that the kinetics of the response to cell wall stressors was slightly different depending on the compound added (Figure S2).

To address whether activation of Mpk1 was dependent on Bck1 or Mkk1, we examined the phosphorylation levels of Mpk1-HA in *bck1*Δ and *mkk1*Δ cells after treatment with 50 µM CFW. No activated form of Mpk1 in either *bck1*Δ or *mkk1*Δ cells was detected (Figure 2B). Thus, both Bck1 and Mkk1 appear to be required for appropriate phosphorylation of Mpk1 *in vivo*.

We also analyzed whether kinase activity of Mpk1 was increased after exposure to CFW. Catalytic activity of Mpk1 was directly monitored in an immunoprecipitation-kinase assay. Cell extracts from cultures treated with 50 µM CFW were prepared, HA-tagged Mpk1 was immunoprecipitated and activity in phosphorylating Myelin Basic Protein (MBP) was determined (Figure 2C). Immunoprecipitated kinase obtained from CFW-treated cells was more active in phosphorylating MBP than that from control non-treated cells.

To determine whether Mpk1 phosphorylation as well as kinase activity was required for *in vivo* activity, two Mpk1 mutant alleles were constructed. First, Mpk1^AEF^ contains mutations (T193A, Y195F) in the conserved phosphoacceptor site and should therefore be unable to be phosphorylated at the conserved TEY motif. Second, Mpk1^KD^ contains a single mutation (K55R) in the ATP-binding pocket and should therefore produce a kinase-dead mutant protein (Figure 2D). These mutant alleles were also engineered to produce HA-tagged variants. By immunoblotting we found no evidence for phosphorylation using the phospho-epitope specific MAPK antibody with proteins extracted from the strain expressing the Mpk1^AEF^ variant. In contrast, the Mpk1^KD^ mutant protein showed a phosphorylation signal that was comparable to wild-type levels (Figure 2E). When kinase activity was measured by immunoprecipitation, we found that both immunoprecipitated Mpk1^KD^ and Mpk1^AEF^ were strongly reduced in the capacity to phosphorylate MBP (Figure 2F). Finally, we asked whether cells carrying these mutant alleles were more sensitive to CFW and found this to be the case (Figure 2G). Taken together, these results indicate that kinase activity of Mpk1 is dependent on T-loop phosphorylation, which is required for appropriate response to cell wall damage.

In summary, these results support the notion that the Mpk1, Mkk1 and Bck1 kinases are *bona fide* core elements of the CWI pathway of *U. maydis*.

### Over-activation of CWI pathway inhibits growth in *U. maydis*

**Figure 3:**
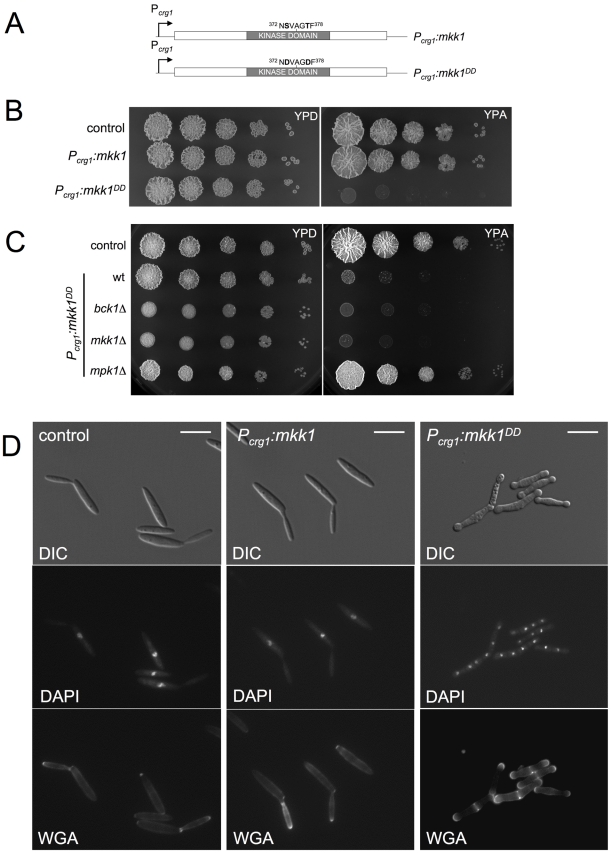
Expression of an activated Mkk1 allele is toxic in *U. maydis*. (A) *mkk1* and *mkk1^DD^* alleles under the control of P*crg1* are shown schematically. Residues mutated are shown at the top. (B) Growth ability of cells expressing an ectopic copy of *mkk1* and *mkk1^DD^* alleles was examined by spotting serial dilutions of exponential cultures of UMA10 (control), UMA12 (*P_crg1_:mkk1*) and UMA7 (*P_crg1_:mkk1^DD^*) strains in rich medium with either 2% glucose (YPD, repressing conditions) or 2% arabinose (YPA, inducing conditions). Plates were incubated at 28°C. (C) Toxicity is suppressed by deletion of gene encoding downstream kinase but not by upstream kinase. Dilution of cell cultures from UMA10 (control), UMA7 (*P_crg1_:mkk1^DD^* wt), UMA57 (*P_crg1_:mkk1^DD^ bck1*Δ), UMA9 (*P_crg1_:mkk1^DD^ mkk1*Δ) and UMA13 (*P_crg1_:mkk1^DD^ mpk1*Δ) were treated as above. (D) Morphology of cells expressing *mkk1^DD^*. YPA cultures of UMA10 (control), UMA12 (*P_crg1_:mkk1*) and UMA7 (*P_crg1_:mkk1^DD^*) were incubated at 22°C (to delay massive cell lysis, see text) for 8 h. Bar 15 µm.

We wished to examine the consequences of over-activation of CWI pathway in *U. maydis*. To approach this issue and to avoid possible side effects on growth by the presence of cell wall stressors known to activate CWI pathway in *U. maydis*, we generated a conditionally activated allele of Mkk1. MEKs are activated by phosphorylation of two Ser/Thr residues located between the kinase subdomains VII and VIII [39], [40]; and previous studies have shown that mutation of these residues to Asp results in constitutive activation [41], [42]. Therefore we generated Mkk1^DD^ in which the conserved Ser373 and Thr377 residues were both replaced with Asp. We constructed an integrative plasmid in which the *mkk1^DD^* allele was under the control of the *crg1* promoter [43]. Thus, expression of *mkk1^DD^* can be achieved by inducing *crg1* with the addition of arabinose to the culture medium. We also constructed a control plasmid carrying the wild-type allele under *crg1* control (Figure 3A). A single copy of either construct or the empty vector was inserted at the *ip* locus in wild-type cells [44]. We found that under conditions that promoted expression of *crg1* promoter (medium containing arabinose as carbon source, YPA) the growth of the strain carrying the activated allele was severely impaired on solid medium (Figure 3B). This inhibitory effect was rescued by loss of function of the downstream kinase *mpk1* but not by the upstream kinase *bck1* (Figure 3C).

When the *crg1* promoter was repressed, cells carrying *mkk1^DD^* were morphologically indistinguishable from control strains (not shown). When induced, cells expressing the ectopic copy of *mkk1^DD^* allele had a strong lysis phenotype as evidenced by the frequent presence of cell “ghosts” and cell debris (Figure S3). Lysis could be largely delayed by growing the cells at low temperature (22°C, unpublished observations). Therefore, we analyzed the morphological effects of expression of *mkk1^DD^* growing cells at 22°C. Under this condition, ectopic expression of the wild-type copy of *mkk1* had no effect on cell morphology (Figure 3D, middle column). Expressing *mkk1^DD^* resulted in aberrant morphology, featuring cell aggregates containing several septa that delimited cell compartments, each carrying a single nucleus (Figure 3D, right column). In addition, there was swelling of the tips of these cell aggregates and these swollen areas stained strongly with FITC-WGA, indicating a continuous deposition of cell wall material at the tips (Figure 3D). These morphological defects were suppressed when *mkk1^DD^* was expressed in cells deleted of *mpk1* (Figure S4).

**Figure 4:**
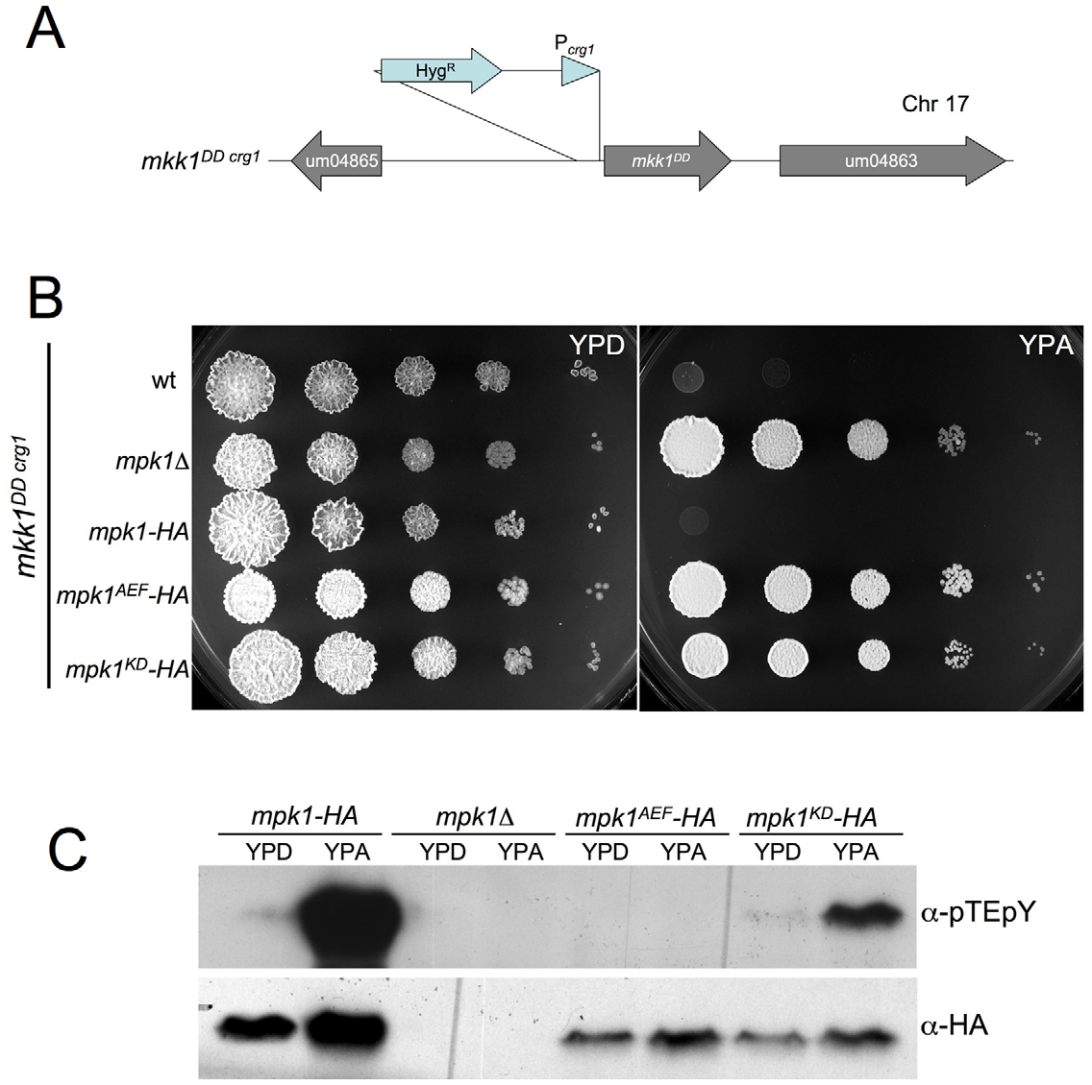
Expression of *mkk1^DD^* overactivates Mpk1. (A) Scheme of the conditional allele *mkk1^DDcrg1^*. (B) Toxicity after *mkk1^DD^* expression is suppressed by *mpk1* alleles defectives in T-loop phosphorylation or kinase activity. Dilution of cell cultures from UMA20 (control, wt), UMA42 (*mpk1*Δ), UMA43 (*mpk1-HA*), UMA64 (*mpk1^AEF^-HA*) and UMA65 (*mpk1^KD^-HA*) were treated as in Figure 3. Plates were incubated at 28°C. (C) T-loop phosphorylation of above strains. Cells were incubated in the indicated media for 8 h at 22°C.

To obtain further support for the idea that it was the activation of the Mpk1 kinase activity that was responsible of these inhibitory effects, we replaced the endogenous *mkk1* locus with the constitutively activated *mkk1^DD^* allele under the control of *crg1* (*mkk1^DDcrg1^* allele, Figure 4A) in strains harboring either the HA-tagged *mpk1* allele or its derivates *mpk1^AEF^-HA* and *mpk1^KD^-HA*. Growth impairment after expression of *mkk1^DD^* allele was suppressed when the downstream Mpk1 kinase was either unphosphorylatable due to mutation at the conserved TEY motif or was disabled for kinase activity (Figure 4B). Moreover, with expression of the *mkk1^DD^* allele there was a dramatic increase in the level of Mpk1 phosphorylation (Figure 4C).

These results are consistent with a model in which Mpk1 is activated by Mkk1, and its over-activation results in inhibitory effects on *U. maydis* cell growth.

### Over-activation of Mpk1 forces cells to escape from G2 phase in *U. maydis*

**Figure 5:**
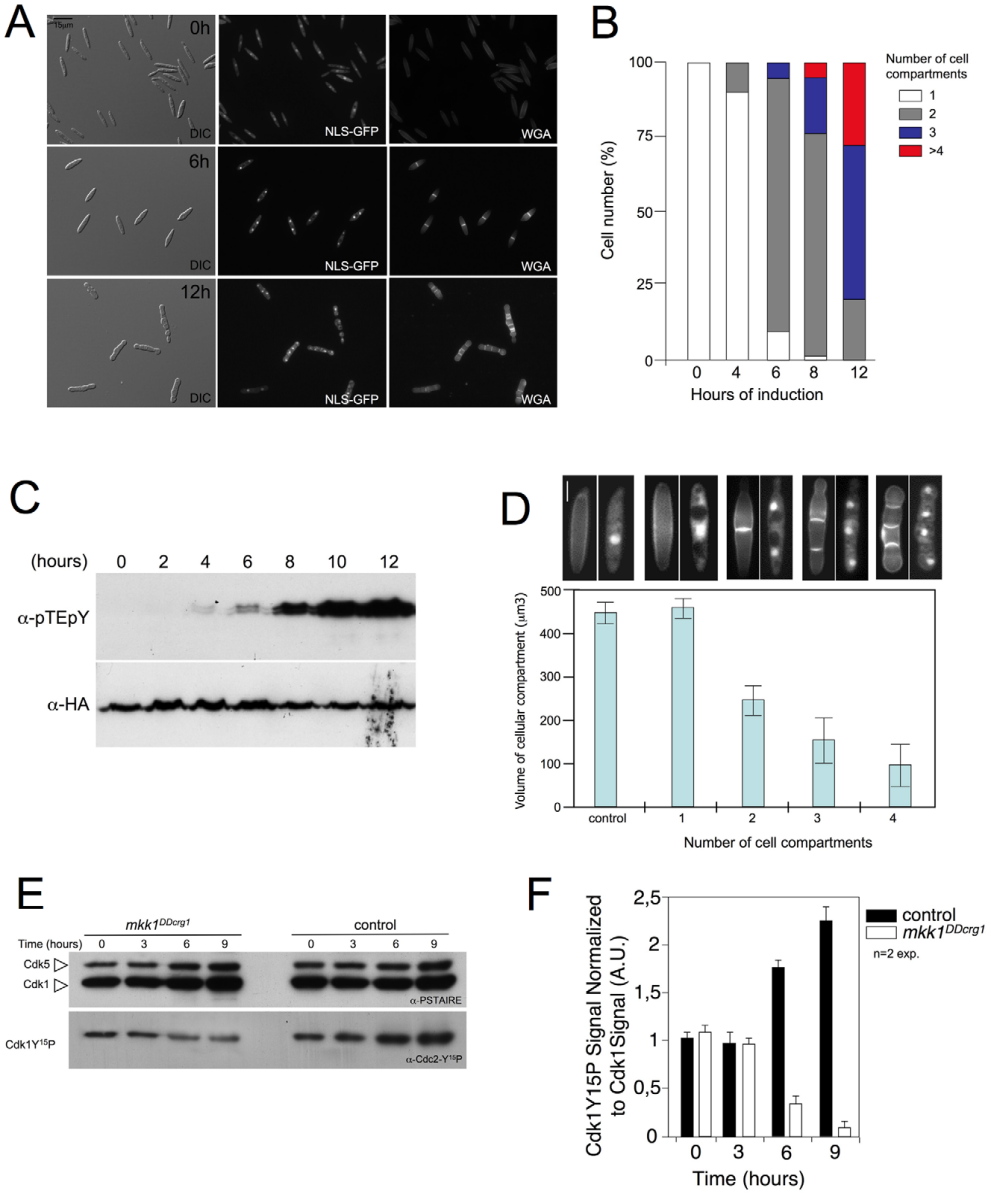
Over-activation of Mpk1 resulted in shortened G2 phase. (A) Time-course of morphological changes induced after *mkk1^DD^* expression. UMA54 cells carrying a NLS-GFP fusion to detect nuclei as well as a HA-tagged *mpk1* allele, were incubated in YPA at 22°C along 12 h. Samples were withdrawn at different times and submitted, after WGA-TRITC staining, to microscopic observation. Bar 15 µm. (B) Quantification of the number of cell compartments per cell aggregate at different time points. At least 50 cell aggregates were counted at each time point (2 independent experiments). (C) Time-course of T-loop phosphorylation of Mpk1 after *mkk1* ^DD^ induction. (D) Size of cell compartments decreases after each mitosis. Volume of each cell compartment (assuming each cell compartment was a cylinder) was obtained after measurement of length and wide of 30 cell compartments in each cell type (2 independent experiments). At the top, cell images at same magnification (Bar 5 µm) of the four cell types measured. Cells were stained with WGA-TRITC to detect septa. Mean and s.d. are shown. (E) Levels of Cdk1 inhibitory phosphorylation after *mkk1^DD^* expression. Protein extracts from FB1 wild-type cells (control) or UMA20 (*mkk1^DDcrg1^*) growing in inducing conditions (YPA) for the indicated time (hours) were separated by SDS-PAGE. Immunoblots were incubated successively with an antibody that recognizes the Cdk1 phosphorylated form (anti-Cdc2-Y^15^P) and anti-PSTAIRE, which recognizes Cdk1 as well as Cdk5. (F) Levels of Cdk1 phosphorylation were determined by quantifying the level of antibody signal using a ChemiDoc (Bio-Rad). Signal from the phosphopeptide-specific antibodies was normalized to the amount of phosphorylation of control strain at time zero. Differences in loading of samples were corrected by dividing each phosphopeptide-specific antibody signal by the Cdk1 (anti-PSTAIRE) antibody signal. Mean and s.d. are shown (n = 2 exp.).

To characterize the morphogenetic defects caused by the over-activation of Mpk1 further, we monitored the morphological appearance of nuclei and cell septa. For nuclear staining we expressed GFP fused with a nuclear localization signal (NLS) and for septa, we used WGA-TRITC for visualization. Cells carrying the *mkk1^DDcrg1^* allele, as well as the *mpk1-HA* allele and constitutively expressed NLS-GFP [45], were incubated at inducing conditions for 12 h. Samples were obtained at different time points and used for Western analysis (to asses the Mpk1 activation level, Figure 5C) as well as for microscopic observations (to asses nuclear number and the presence of septa after WGA-TRITC staining, Figure 5A). Four hours after induction, cells started to divide via a central septum, and this correlated with the presence of activated Mpk1 kinase. By 6 h induction more than 75% of the cells showed two cell compartments and at this time a clear activated Mpk1 signal was apparent. Further incubation (8 h) resulted in the appearance of cell aggregates (septa-mediated division produced cell aggregates due to the lack of cell separation) composed of three or more cell compartments, each carrying one nucleus. Finally, after 12 h of incubation more than 80% of the cells showed three or more cell compartments (Figure 5B).

An interesting observation was that the size of cell compartments appeared to decrease after each cell division. We quantified volume in each cell compartment by measuring length and width of each, and deduced that the volume of each cell compartments decreased as the number of cell compartments per aggregate increased (Figure 5D). In *U. maydis*, cell size in daughter cells is dependent on the length of G2 phase: the longer the G2 phase, the larger the daughter cell and *vice versa* [13]. Previous reports [46], [47] indicated that shortening of G2 phase by decreasing the levels of Cdk1 inhibitory phosphorylation at Tyr15 causes a decrease in the size of daughter cells as well as abolition of the budding program and the division by inserting septa. This change in the division mode is also accompanied by the inhibition of cell separation, resulting in cell aggregates. In fact, alteration of the levels of cell cycle regulators affecting Cdk1 inhibitory phosphorylation such as overproduction of Cdc25 phosphatase or down-regulation of Wee1 kinase [46], [47] resulted in the formation of cell aggregates that were reminiscent of those observed after over-activation of Mpk1. Therefore, we analyzed levels of Cdk1 inhibitory phosphorylation upon over-activation of Mpk1. For this we used a specific antibody raised against the Tyr15 phosphorylated human Cdc2 peptide (Y^15^P), which also recognizes the Tyr15-phosphorylated form of *U. maydis* Cdk1 [47]. We found a decrease of around 9-fold in the levels of Tyr^15^P-Cdk1 when the *mkk1^DDcrg1^* allele was expressed (Figure 5E and 5F).

Since the observed decrease in inhibitory phosphorylation could promote the escape from G2 phase, and as a consequence a reduction in the size of daughter cells as well as production of cell aggregates, we sought to analyze what was the percentage of cells in G2 phase in conditions of *mkk1^DDcrg1^* allele expression. For this we took advantage of previous studies showing that the mitotic cyclin Clb2 is synthesized during G2 phase and degraded at anaphase [48]. We constructed a strain carrying a *clb2-GFP* fusion at its native locus as well as the *cut11-RFP* fusion [49] to localize nuclear membrane and confirmed that the presence of GFP signal correlated with G2 phase (Figure S5). Cells carrying *clb2-GFP* marker and expressing *mkk1^DDcrg1^* allele were scored as being in G2 phase or not, based on the presence or absence of a nuclear GFP signal. We observed that while in the control strain the percentage of cells being in G2 remained high during the incubation period, in the strain expressing *mkk1^DDcrg1^* allele it was obvious a dramatic decrease in the number of cells showing *clb2-GFP* signal (Table 1). In summary these data indicated that overactivation of Mpk1 seems to force cells to escape from G2 phase.

**Table 1:** Cell population showing the G2 marker *clb2-GFP.*

Induction time (h)	Cells with nuclear GFP signal* (%)	
	control	mkk1^DDcrg1^
0	30.4	29.2
3	28.6	18.4
6	32.1	6.7
9	26.4	4.4
12	22.3	1.8

* UMA88 (control) and UMA89 (*mkk1^DDcrg1^*) strains were incubated in YPA for the indicated time and around 100 cells in each time point (n = 2 experiments) were scored for the presence of Clb2-GFP signal. Numbers indicated percentage of cells in G2 phase.

### G2 escape after over-activation of Mpk1 is dependent on Cdc25 phosphatase

**Figure 6:**
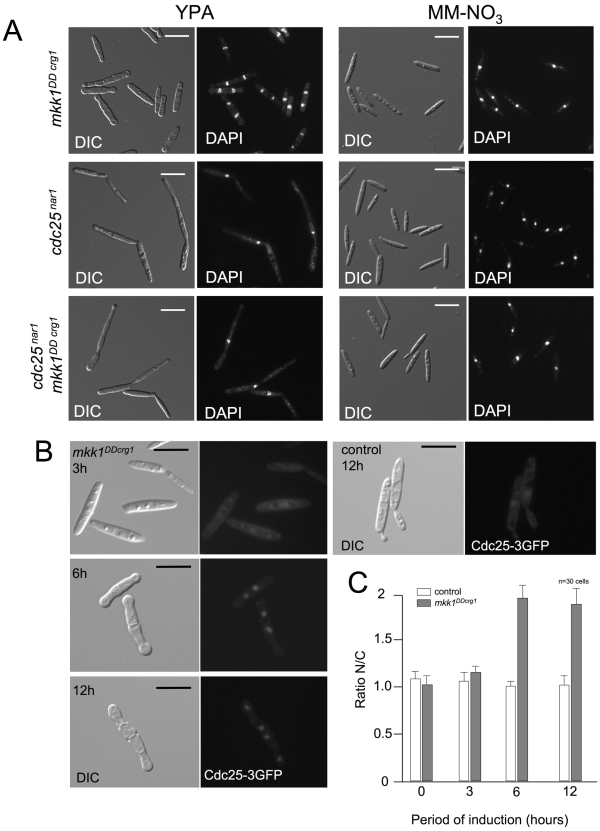
Cdc25 is required for Mpk1-activated premature G2/M transition. (A) Cdc25 is required for Mpk1-induced premature G2/M transition. DIC and DAPI images of UMA20 (*mkk1^DDcrg1^*), UMC27 (*cdc25^nar1^*) and UM56 (*cdc25^nar1^ mkk1^DDcrg1^*) cells grown for 6 h in YPA at 22°C (induction conditions for *crg1*, repressive for *nar1*) or minimal medium amended with nitrate and glucose (MM-NO3; repressing conditions for *crg1* and induction for *nar1*). Bar 15 µm. (B) Cdc25 concentrates in the nucleus upon Mpk1 over-activation. DIC and epifluorescence images of MUM72 cells (control) carrying an endogenous Cdc25-3GFP as well as its derivated UMP184, expressing *mkk1^DD^* (*mkk1^DDcrg1^*), at different time points (hours) of incubation in YPA at 22°C. Bar 10 µm. (C) Graph indicates the quantification of nucleus/cytoplasm ratio of GFP fluorescence signal. Quantification of fluorescence signal was performed by measuring pixel intensity in the nuclei and in an equivalent area of the cytoplasm. Mean and s.d. are shown. 30 cells were measured in each time point (2 independent experiments).

Levels of Cdk1 inhibitory phosphorylation in *U. maydis* are dependent on the activity of Wee1 kinase [47]. We found that over-activation of Mpk1 can overcome high levels of Wee1 produced ectopically (Figure S6). Two possible explanations could account for this result: either Mpk1 over-activation inhibits Wee1 activity or Mpk1 over-activation leads to an increase of some phosphatase activity able to remove inhibitory phosphorylation from Cdk1 -including conditions of high levels of Wee1. In *U. maydis*, the levels of Cdk1 inhibitory phosphorylation also depends on the activity of the Cdc25 phosphatase, which reverses the CDK inhibition [46]. To determine whether the Mpk1-induced escape from G2 was dependent on Cdc25, we introduced the conditional allele *cdc25^nar1^* [46] into a strain carrying the *mkk1^DDcrg1^* allele. This conditional allele enables the expression of *cdc25* in cells incubated in minimal medium plus nitrate as nitrogen source (MM-NO_3_) as well as the down-regulation of *cdc25* expression in rich medium. When cells carrying the *cdc25^nar1^* conditional allele were grown in rich medium (repressive conditions) and in the presence of arabinose (inducing conditions for *mkk1^DDcrg1^*), they arrested in G2 phase (large buds, single nucleus) whether or not the *mkk1^DDcrg1^* allele was expressed, indicating that Cdc25 was necessary for Mpk1-induced G2 escape (Figure 6A). This result indicates that no “alternative” phosphatase, putatively activated by Mpk1, is supplanting the Cdc25 role to promote the observed G2 escape; and also that it is unlikely that Wee1 is the target (direct or indirect) of Mpk1 overactivation: If the activity of Wee1 had been strongly inhibited, then the observed escape from G2 would not require Cdc25 activity. However, we found the opposite result: Cdc25 is required for Mpk1-mediated G2 escape. Therefore, the most plausible explanation is that Cdc25 is activated (direct or indirectly) by the CWI pathway over-activation.

We tried to address the organizational level at which Cdc25 is regulated after Mpk1 over-activation. Western blot analysis of Cdc25 levels in a strain carrying a myc-tagged Cdc25 [46] as well as the *mkk1^DDcrg1^* allele showed no changes in Cdc25 levels after Mpk1 over-activation (not shown). Previous work done in our laboratory established that conditions resulting in nuclear accumulation of Cdc25 correlated with accelerated G2/M transition in *U. maydis* [50], [51]. Therefore, we also analyzed whether Mpk1 over-activation correlated with nuclear accumulation of Cdc25. To address this possibility we monitored the Cdc25 nucleus/cytoplasm distribution in cells carrying the *mkk1^DDcrg1^* allele and expressing GFP-tagged Cdc25 (Figure 6B). To assess changes in localization quantitatively, fluorescence intensities of the Cdc25 signal were determined for a circular region corresponding to the nucleus (N) and a comparable area in the cytoplasm (C) as described [50]. We found under induction conditions (YPA) the ratio N/C in control cells was about 1, while the ratio in cell expressing the *mkk1^DDcrg1^* allele was around 2 (Figure 6C). This correlation would suggest that the nuclear accumulation of Cdc25 is one possible cause for G2 escape when Mpk1 is over-activated.

### N-terminal extension of Cdc25 is important for the response to cell wall damage

**Figure 7:**
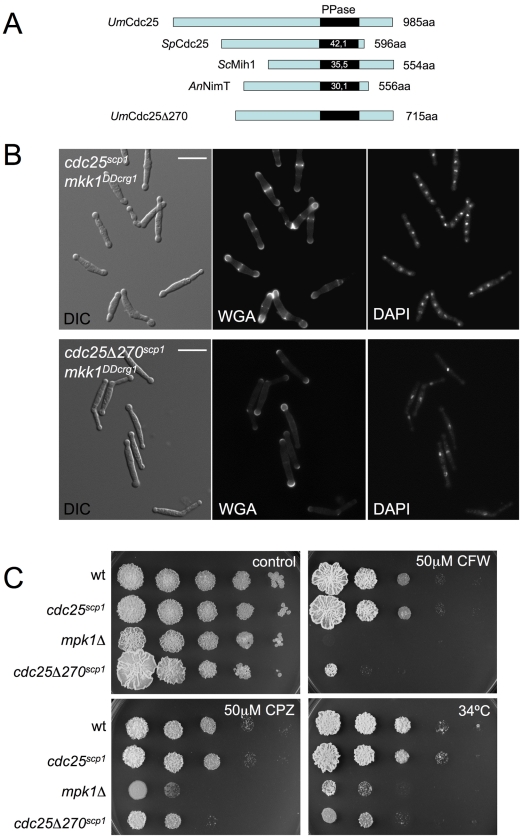
The N-terminal end of Cdc25 is required for Mpk1-induced premature G2/M transition. (A) Representation of the *U.* maydis Cdc25 protein in relation to other fungal Cdc25-like proteins. The percentages inside each box represent the sequence identity of catalytic phosphatase domain when compared to the *U. maydis* sequence. Note the extended N-terminal end of *Um*Cdc25. Derivate lacking the first 270 amino acids is also shown. (B) Cells carrying a *cdc25* allele lacking the first 270 amino acids do not accelerate G2/M transition upon Mpk1 activation. Images show DIC as well as DAPI and WGA-FITC stained cells from UMA62 (*cdc25^scp1^ mkk^DDcrg1^*) and UMA63 (*cdc25*Δ*270^scp1^ mkk^DDcrg1^*) cultures in YPA for 8 h at 22°C. Bar 15 µm. (C) Cells lacking the N-terminal extension of Cdc25 were less resistant to cell wall stressors. Tenfold serial dilutions of cultures of FB1 (wt), UMA61 (*cdc25^scp1^*), UMA3 (*mpk1*Δ) and UMA79 (*cdc25*Δ*270^scp1^*) were spotted in solid YPD medium amended with Calcofluor White (CFW) and Chlorpromazine (CPZ). Plates were grown for 4 days at 28°C (control, CFW and CPZ) or 34°C.

In comparison with other fungal Cdc25-like phosphatases, *U. maydis* Cdc25 carries an unusually long N-terminal extension of around 300 amino acids with no similarity to orthologues in the database (Figure 7A). Previous attempts to ascribe a role to this domain were not definitive because its deletion seemed not to affect Cdc25 function in *U. maydis* growing under normal conditions [46]. We wondered whether this region might be related to the G2 escape in response to Mpk1 over-activation. To address this question we constructed a N-terminal deletion that removed the first 270 amino acids, and we cloned the resulting deletion allele as well as a full-length allele under the control of the constitutively expressed *scp1* promoter, that transcribes at a similar rate as the native *cdc25* promoter (unpublished observations). The alleles *cdc25^scp1^* and *cdc25*Δ*270^scp1^* were exchanged with *cdc25* locus in cells carrying *mkk1^DDcrg1^*. We found that deletion of the extra N-terminal domain of Cdc25 precludes the morphological effects of G2 escape. After 12 h of incubation under inducing conditions the majority of cells carried a single nucleus and these were able to divide by budding. Other effects of Mpk1 over-activation such as the swelling at the tips (Figure 7B) or the inability to form colonies in solid medium (not shown) were unaffected.

To investigate the relationship between an impaired escape from G2 and cell resistance to cell wall stressors, we introduced the *cdc25*Δ*270^scp1^* allele in wild-type cells and measured growth in the presence of cell wall stressors (Figure 7C). We found that cells carrying the *cdc25*Δ*270^scp1^* allele were more sensitive than wild-type cells and control (*cdc25^scp1^*) cells, although to a lesser degree than *mpk1*Δ mutants, suggesting that cell cycle adjustment is one part of the adaptation of cells to these harsh conditions.

### Cell cycle was not accelerated upon CWI pathway activation

**Figure 8:**
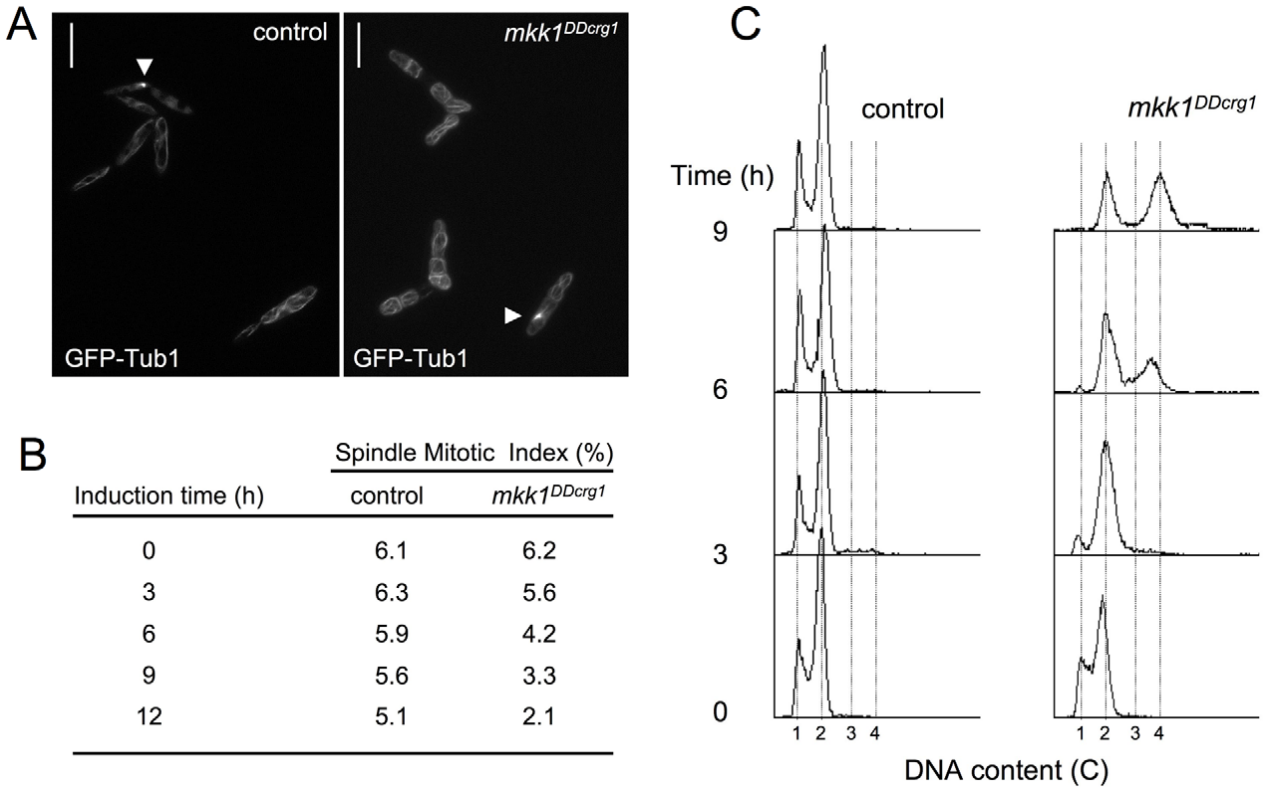
Cell cycle was not accelerated upon CWI pathway activation. (A) Cell images of control (UMP61) and *mkk1^DDcrg1^*-derived (UMA84) cells carrying GFP-Tub1 to detect the microtubule cytoskeleton after 9 h of growth in YPA. Cells in interphase showed microtubules forming a cytoplasmic network, while in mitotic cells the microtubules are concentrated in the spindle (arrowhead). Bar: 15 µm. (B) Spindle Mitotic Index of control cells as well as cells with over-activated Mpk1. Cells were incubated in YPA for the indicated time and around 300 cells in each time point (n = 3 experiments) were scored for the presence of mitotic spindle. Numbers indicated percentage of cells showing mitotic spindle. (C) FACS analysis of the DNA content of FB1 (control) and UMA20 (*mkk1^DDcrg1^*) strains growing in YPA. The period of incubation in testing media is indicated (hours).

The above results indicated that CWI pathway over-activation resulted in an escape from G2 phase as a consequence of the decrease in Cdk1 inhibitory phosphorylation, most likely upon Cdc25 activation. Strikingly, this cell cycle adjustment seems to be necessary for appropriate response of *U. maydis* cells to cell wall stress. The general view is that in fungi, response to stress stimuli often involves a cell cycle arrest in order for cells to adapt to unfavorable stress conditions. However, escape from G2 -without any compensatory change in other phases of cell cycle- would result in a substantial decrease in the generation time (i. e., cell cycle acceleration). To address whether cell cycle was accelerated in response to CWI pathway over-activation, we estimated the doubling time of cells after Mpk1 over-activation. In control wild-type cells growing in YPA the doubling time changed from 2.2 to 2.5 h during the measured interval (12 h of incubation). However, in same conditions cells expressing the *mkk1^DDcrg1^* allele showed a doubling time of around 3.2 h during the first cycle (cells carrying one single nucleus to cell aggregates showing two nuclei), while the second cycle (cell aggregates carrying two nuclei to cell aggregates carrying 4 nuclei) was roughly 6.4 h. As this result suggests that upon Mpk1 over-activation the cell cycle is slowing, we analyzed the proportion of cells entering into mitosis. Cells carrying a GFP-a tubulin fusion were scored as being in mitosis or not being in mitosis based on the presence or absence of a mitotic spindle (Spindle Mitotic Index, SMI; Figure 8A). With the observed increase in the duplication time, we consistently found a lower SMI in cells expressing the *mkk1^DDcrg1^* allele (Figure 8B). In summary these data indicated that in spite of the apparent shorter G2 phase cells expressing the *mkk1^DDcrg1^* allele showed a more lengthy cell cycle than control cells.

We believe that a plausible explanation for the above apparently contradictory results is that escape from G2 phase is compensated by the lengthening at another cell cycle phase (i.e. G1 or S-phase). To analyze whether G1 or S-phase was affected by over-activation of CWI pathway, we analyzed the DNA content of cells at different time points upon induction of *mkk1^DDcrg1^* allele using Fluorescence/Activated Cell Sorter (FACS) (Figure 8C). We observed that upon over-activation of Mpk1 kinase, cells (or cell aggregates) accumulated with a 2C DNA content and over time even higher (3-4C). Since during that period the majority of cell aggregates carried two nuclei (for instance, after 6 h upon induction around 70% of cells showed a 2C DNA content and also showed 2 cell compartments, Figure 5B) we can deduce that these nuclei have a 1C DNA content each and therefore most likely are in G1 phase.

Taken together, these results suggest that upon over-activation of the CWI pathway, the cell cycle is affected in at least two different ways. Cells seem to escape from G2 phase and G1-S transition appears to be dramatically delayed.

### Cell wall stressors affect cell cycle in wild-type cells

**Figure 9:**
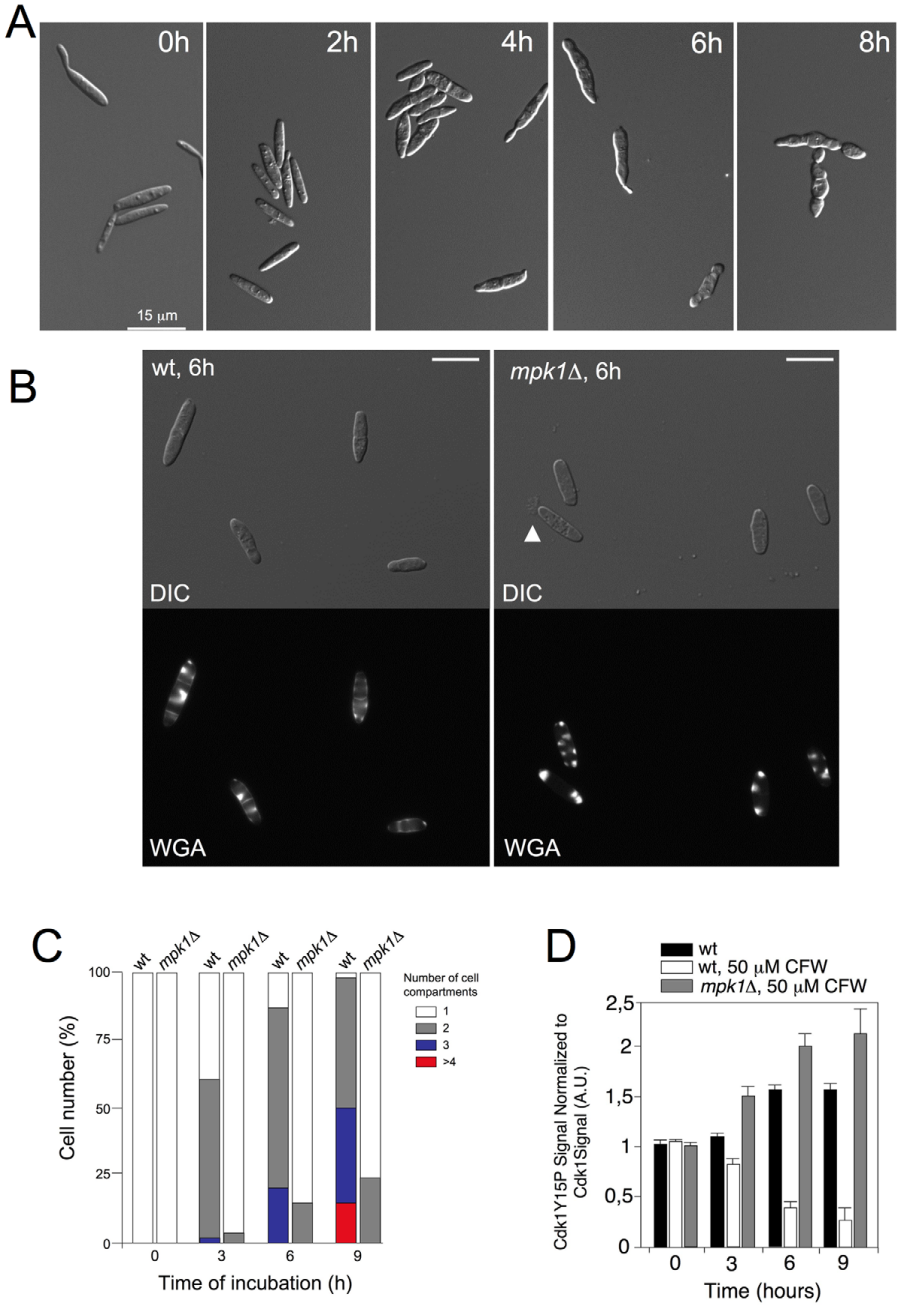
Cell cycle changes after CFW treatment. (A) DIC images of wild-type (FB1) cells treated treated with 50 µM CFW for the time indicated. Bar: 15 µm. (B) Aspect of wild-type and *mpk1*Δ cells grown in the presence of 50 µM CFW for 6 hours. WGA-FITC staining shows the presence of cell compartments in wild-type cells as well as its absence in mutant cells. Arrowhead indicates a cell leaking cytoplasmic material at the tip. Bar: 10 µm. (C) Quantification of the number of cell compartments per cell aggregate at different time points in wild-type and *mpk1*Δ cells. At least 50 cell aggregates were counted at each time point (2 independent experiments). (D) Levels of Cdk1 inhibitory phosphorylation after CFW treatment in control (FB1) and *mpk1*Δ (UMA3) cells. Cdk1 inhibitory phosphorylation was measured as described in Figure 5. Mean and s.d. are shown (2 independent experiments).

One concern in our approach is the use of a constitutive activated form of MEK. We reasoned that if our above results reflect a physiological response we should to be able to observe a similar response in wild-type cells under cell wall stress and that response should be abolished in a CWI pathway-defective mutant. Consistently, we observed a morphological response reminiscent of that observed with the *mkk1^DDcrg1^* allele when wild-type cells were treated with 50 µM CFW: Cell aggregates resulted from septation without cell separation (Figure 9A–9C). When *mpk1*Δ cells were treated with CFW, cells remained undivided, WGA-FITC accumulation was observed around cell surface, and eventually these cells lysed near the cell tip (Figure 9B, arrowhead). In wild-type cells these results also correlated with a decrease in the levels of Cdk1 inhibitory phosphorylation after treatment with CFW, and again this response was dependent on an active CWI pathway (Figure 9D). We also found that the Spindle Mitotic Index of wild-type cells decreased to 3.5 after 6 hours of CFW while in untreated cells it was of 5.6 (no measurements were taken in *mpk1*Δ mutants because microtubules were severely disordered in these cells after CFW treatment). In summary, these results mirrored those found when a constitutively activated *mkk1^DDcrg1^* allele was used.

## Discussion

The main conclusion of this study is that in *U. maydis* activation of the CWI pathway promotes escape from G2 phase. This result contrasts with the G2 cell cycle arrest that occurs in *S. cerevisiae* in response to CWI pathway activation [12]. Since our results strongly suggest that the pathway described in this study is the *bona fide* CWI pathway in *U. maydis*, it is of interest to understand what might explain the disparate responses in these organisms. This distinct pattern of cell cycle arrest likely reflects different cellular tactics used to pursue a common strategic mechanism. Cell wall is remodeled throughout the cell cycle. During periods of polarized cell growth, the wall is loosened by digestive enzymes, such a chitinases and glucanases, and expanded at these specific regions on the cell surface. During these periods, cells experience the greatest wall stress and are most vulnerable to lysis. Several reports support the view that apical growth is exquisitely sensitive to loss of integrity –tip lysis occurs in response to osmotic stress or treatment with drugs targeting chitin and glucan synthases such as nikkomycin and caspofungin [52], [53]. It follows that phases where polar growth is most active are the phases in which cells are most vulnerable to cell wall instability.

Under these conditions, if for some reason cell wall biosynthesis is compromised, a survival strategy might be to escape from cell cycle phases where polar growth is higher. Interestingly, the cell cycle stage at which polar growth is maximum is different in each organism. While in *S. cerevisiae* polar growth reaches its maximal at late G1 and switches to isotropic growth during G2 [54], in *U. maydis* the maximum of polar growth occurs at the G2 phase where the characteristic elongated bud is made [55]. Consistently, activation of CWI pathway in *S. cerevisiae* drives the cell cycle to remain in G2 phase, where cell growth is isotropic, while in *U. maydis* activation of CWI drives the cell cycle to escape from G2, where polar growth is more pronounced.

Our results also suggest that the promoted escape from G2 does not necessarily imply a faster cell cycle. In fact, we observed a pronounced increase in the generation time upon CWI pathway over-activation, suggesting that shortening of the G2 phase is compensated by the lengthening of another cell cycle phase. We believe that this delay occurs on G1/S transition, although we do not understand the mechanism through which this delay takes place. At this point we do not know whether this G1/S delay is part of the cell cycle response to over-activation of CWI pathway or is simply a consequence of the observed growth defect. Previously reported *U. maydis* G1 regulators such as the APC activator Cru1 [56] or the G1 cyclin Cln1 [57] seem not to affect this delay (NC, unpublished observations). We investigated in more detail the mechanism responsible for the observed G2 escape upon CWI pathway over-activation. It appears that activation of the CWI pathway results in the accumulation of the Tyr15-unphosphorylated form of Cdk1 and most likely it results in the observed escape from G2 towards mitosis. We found that Cdc25 was the sole mitotic-inducer phosphatase in *U. maydis* required for this phenotype. In response to activation of CWI pathway, Cdc25 accumulates in the nucleus, providing a likely explanation for the increase of the unphosphorylated form of Cdk1. We also found that a mutant allele of Cdc25 lacking the first 270 residues ameliorates the premature G2/M transition upon activation of CWI pathway. In addition, these mutant cells showed a higher sensitivity to the presence of cell wall stressors. We hypothesize that Mpk1 directly targets Cdc25 at its extended N-terminal domain promoting nuclear import. Recently, it has been reported in *Xenopus* that p42 MAPK, the *Xenopus* ortholog of mammalian ERK2, is able to directly phosphorylate and activate Cdc25C [58]. To date, however, we have obtained no experimental support for this notion in *U. maydis*. We did not detect any physical interaction between Cdc25 and Mpk1 by co-immunoprecipitation (not shown), nor did we observe any differential migration of Cdc25 in SDS-PAGE gels after response to Mpk1 activation that might suggest direct phosphorylation. We considered alternative explanations. A three-amino acid motif (S/TPS/T) was described that is phosphorylated upon MAPK-associated stimulation to induce Importin 7-dependent nuclear translocation [59]. We were intrigued to note the N-terminal extension of *U. maydis* Cdc25 contains several S/TPS/T motifs. However, deletion of the putative Importin 7 (um01606) had no effect on the MAPK-mediated nuclear translocation of Cdc25 (not shown). We are now investigating other factors that are able to specifically interact with the N-terminal domain of Cdc25, such as 14-3-3 protein [50].

One cautionary aspect of our approach is whether the cell cycle effects observed are a consequence of the toxicity resulting from forced expression of *mkk1^DD^*. We believe that our results provide strong evidence that at least, G2 escape seems to be a result of CWI pathway activation unrelated from the toxicity. First, we were able to observe similar effects on the cell cycle upon treatment of wild-type cells with cell wall stressors and these effects were dependent on the presence of an active CWI pathway. Second, the correlation between inability to escape from G2 and enhanced sensitivity to cell wall stressors observed with the Cdc25 mutant lacking the N-terminal extension, strongly supports the idea that cell cycle modification is part of the cell response to cell wall insults in *U. maydis*. Third, the toxicity effects we observed are unlikely to be related to the cell cycle effects. Cells expressing *mkk1^DD^* had a strong lysis phenotype and the Mkk1^DD^-induced toxicity can be alleviated growing the cells in the presence of an osmotic stabilizer or at low temperature (not shown). However, these conditions do not preclude the premature G2/M transition observed when CWI pathway is activated. Furthermore in a strain carrying the *cdc25*Δ*270* allele, the expression of *mkk1^DD^* was also toxic. We propose that this toxicity could be a consequence of unbalanced cell wall synthesis because of the sustained high activation of the CWI pathway. Concomitant, but independent of G2 escape, was the dramatic swelling of cell tips that stained strongly with WGA, indicating a continuous deposition of new cell wall material occurring at the tip. It is worth noting that toxicity was also reported in *S. cerevisiae* upon expression of an activated *MKK1* allele [60], in spite of the different effects on cell cycle that activation of CWI pathway has in these species.

In summary, our findings support the view that in response to environmental input, the cell has to choose which cell cycle stage is the most appropriate cellular environment to respond to such stimulus and then moves towards such a cell cycle phase. The appropriate cell cycle stage could well differ from one organism to other, and so the observed response.

## Materials and Methods

### Strains and growth conditions

*Ustilago maydis* strains are listed in Table 2 and are derived from FB1 background [61]. Media were prepared as described [62]. Controlled expression of genes under the *crg1* and *nar1* promoter was performed as described previously [48], [63]. FACS analyses were described previously [22]. Drug sensitivity assays were performed as described [64].

**Table 2:** *U. maydis* strains used in this study.

Strain	Relevant genotype	Reference
FB1	*a1 b1*	[61]
UMA1	*a1 b1 bck1*Δ	This work
UMA2	*a1 b1 mkk1*Δ	This work
UMA3	*a1 b1 mpk1*Δ	This work
UMA4	*a1 b1 bck1*Δ *mkk1*Δ	This work
UMA5	*a1 b1 bck1*Δ *mpk1*Δ	This work
UMA6	*a1 b1 mkk1*Δ *mpk1*Δ	This work
UMA8	*a1 b1 bck1*Δ *mkk1*Δ *mpk1*Δ	This work
UMA44	*a1 b1 mpk1-3HA*	This work
UMA44.2	*a1 b1 mpk1-3HA bck1*Δ	This work
UMA44.3	*a1 b1 mpk1-3HA mkk1*Δ	This work
UMA59	*a1 b1 mpk1^AEF^-3HA*	This work
UMA60	*a1 b1 mpk1^KD^-3HA*	This work
UMA10	*a1 b1 P_crg1_*	This work
UMA12	*a1 b1 P_crg1_:mkk1*	This work
UMA7	*a1 b1 P_crg1_:mkk1^DD^*	This work
UMA57	*a1 b1 P_crg1_:mkk1^DD^ bck1*Δ	This work
UMA9	*a1 b1 P_crg1_:mkk1^DD^ mkk1*Δ	This work
UMA13	*a1 b1 P_crg1_:mkk1^DD^ mpk1*Δ	This work
UMA20	*a1 b1 mkk1^DD crg1^*	This work
UMA42	*a1 b1 mkk1^DD crg1^ mpk1*Δ	This work
UMA43	*a1 b1 mkk1^DD crg1^ mpk1-3HA*	This work
UMA64	*a1 b1 mkk1^DD crg1^ mpk1^AEF^-3HA*	This work
UMA65	*a1 b1 mkk1^DD crg1^ mpk1^KD^-3HA*	This work
UMA54	*a1 b1 mkk1^DD crg1^ mpk1-3HA NLS-GFP*	This work
UMC48	*a1 b1 P_crg1_:wee1*	[47]
UMA79	*a1 b1 P_crg1_:wee1 mkk1^DD crg1^*	This work
UMC27	*a1 b1 cdc25^nar1^*	[46]
UMA56	*a1 b1 cdc25^nar1^ mkk1^DD crg1^*	This work
MUM72	*a1 b1 cdc25-3GFP*	[48]
UMP184	*a1 b1 cdc25-3GFP mkk1^DD crg1^*	This work
UMA62	*a1 b1 cdc25^scp^ mkk1^DD crg1^*	This work
UMA63	*a1 b1 cdc25*Δ*270^scp^ mkk1^DD crg1^*	This work
UMA61	*a1 b1 cdc25^scp^*	This work
UMA79	*a1 b1 cdc25*Δ*270^scp^*	This work
UMP61	*a1 b1 GFP-tub1*	[66]
UMA84	*a1 b1 GFP-tub1 mkk1^DD crg1^*	This work
UMA88	*a1 b1 clb2-GFP cut11-RFP*	This work
UMA89	*a1 b1 clb2-GFP cut11-RFP mkk1^DD crg1^*	This work

### Protein analysis

Protein extracts and Western blotting were performed as described previously [65]. To purify Mpk1 complexes and analyze their kinase activity previously described protocols were followed [65]. All quantification was done using a Phosphorimager (Molecular Dynamics). To detect the phosphorylated and non-phosphorylated forms of Cdk1 and Mpk1 commercial antibodies were used as described [47], [48], [65]. Primary antibody was followed by a secondary antibody conjugated to horseradish peroxidase and immunoreactive proteins were visualized using a chemiluminescent susbtrate. The chemiluminescent signal was analyzed using ChemiDoc XCS+ (Molecular Imager, Bio-Rad).

### Plasmid and strain constructions

Plasmid pGEM-T easy (Promega) was used for cloning, subcloning and sequencing of genomic fragments and fragments generated by PCR. Sequence analysis of fragments generated by PCR was performed with an automated sequencer (ABI 373A) and standard bioinformatic tools. To construct the different strains, transformation of *U. maydis* protoplasts with the indicated constructions was performed as described previously [66]. Gene replacement into the corresponding loci was verified by diagnostic PCR and subsequent Southern blot analysis. *U. maydis* DNA isolation was performed as previously described [66]. Fluorescent protein fusions were described previously: Cdc25-3GFP [50], NLS-GFP [45], GFP-Tub1 [55], Cut11-RFP [49], Clb2-GFP (JPM, unpublished).

Deletion of each kinase gene was performed using PCR-based gene targeting [27]. Briefly, a pair of DNA fragments flanking the corresponding kinase ORF were amplified and ligated to antibiotic resistance cassettes via *Sfi*I sites. The 5′ and 3′ fragments were amplified using specific oligonucleotide pairs (Table 3). Each fragment was about 1 kbp in length. For *bck1* we used primer pairs Bck1-2/Bck1-3 (5′ end) and Bck1-4/Bck1-5 (3′ end) and we ligated to a nourseotricin resistance cassette. For *mkk1* we used primer pairs Mkk1-2/Mkk1-3 (5′ end) and Mkk1-4/Mkk1-5 (3′ end) and we ligated to a hygromycin resistance cassette. For *mpk1* we used primer pairs Slt2-4/Slt2-r (5′ end) and Slt2-5/Slt2-f (3′ end) and we ligated to carboxin as well as hygromycin resistance cassettes. Double and triple mutants were obtained after cross the respective parental strains and selection of haploid progeny resistant to the respective markers [62].

**Table 3:** Oligonucleotides used in this study.

Name	Sequence
BCK1-2	5′ACGCACCACGCACGACCACAGACTCGT3′
BCK1-3	5′GTGGGCCATCTAGGCCAACGGTTGGATTGGCTTGA3′
BCK1-4	5′CACGGCCTGAGTGGCCATTGCTTCATGCGGTTGC3′
BCK1-5	5′TCTATGTCCACCAACATGCTCATGGGT3′
MKK1-2	5′GCCTGATGCAAGCAAATCAGTGTCAGT3′
MKK1-3	5′GTGGGCCATCTAGGCCAAGCAGCAGCACATACAGTC3′
MKK1-4	5′CACGGCCTGAGTGGCCCTCACGTGCAATCTTAGTATG3′
MKK1-5	5′CTCAAGCTTTTGTCGTAACGATGGTGT3′
MKK1-E1	5′CATATGGCCTCGCTCATTCCACCGAAG3′
MKK1-E2	5′GAGCTGATCAACGACGTTGCCGGCGATTTTACGGGCACA3′
MKK1-E3	5′TGTGCCCGTAAAAGTACCGGCAACCGAGTTGATCAGCTC3′
MKK1-E4	5′GAATTCAGCAACATAATCAGATTGCAC3′
MKK1-8E	5′GAATTCCGGCGTATACTCATACGCCAT3′
MKK1-9B	5′GGATCCGCCTTTTGACCATCAATCTC3′
SLT2-4	5′AGTCACAACGTGTTGGTTCAAAGTTTA3′
SLT2-r	5′GTGGGCCATCTAGGCCTTGTCTACGTCAAGATCA3′
SLT2-5	5′TTTCATGTGGGAATGGTATACCACCAA3′
SLT2-f	5′CACGGCCTGAGTGGCCACAGCTCACCTCGCGGTC3′
SLT2-KF	5′GGTACCACTCCCGACTGTACAGTGCAGTA3′
SLT2-10	5′GCTGATCTTGACGTAGACCATATGTCGGCACATCCATCAAAC3′
SLT2-11	5′GTTTGATGGATGTGCCGACATATGGCTTACGTCAAGATCAGC3′
SLT2-KR	5′GGTACCGATTTTGACCGCGAGGTGAGCTG3′
SLT2-A1	5′GGCGCAGGTTTCATGGCAGAATTCGTAGCAACTCGATGG3′
SLT2-A2	5′CCATCGAGTTGCTACGAATTCTGCCATGAAACCTGCGCC3′
SLT2-B1	5′GAAAGCGTAGCTATCAGAAAGATCACCAACGTC3′
SLT2-B2	5′GACGTTGGTGATCTTTCTGATAGCTACGCTTTC3′

For N-terminal fusion of Mpk1 with HA, a *Nde*I site was introduced at the ATG of *mpk1* locus using PCR-based two-step mutagenesis protocol (amplification was performed using the primer pairs: Slt2-KF/Slt2-11 and Slt2-10/Slt2-KR). A stop-less *Nde*I 3xHA cassette was introduced at the respective *Nde*I site and the HA-Mpk1 fusion integrated by homologous recombination under their respective native *mpk1* promoter.

The mutant *mpk1^AEF^* allele was constructed by the assembly of two fragments carrying the T193A and Y195F mutations generated by PCR with the primer pairs Slt2-KF/Slt2-A2 and Slt2-A1/Slt2-KR. As template we used the *mpk1-HA* allele.

The mutant *mpk1^KD^* allele was constructed by the assembly of two fragments carrying the K55R mutation generated by PCR with the primer pairs Slt2-KF/Slt2-B2 and Slt2-B1/Slt2-KR. As template we used the *mpk1-HA* allele.

The mutant *mkk1^DD^* allele was constructed by the assembly of two fragments carrying the S373D and T377D mutations generated by PCR with the primer pairs Mkk1-E1/Mkk1-E3 and Mkk1-E2/Mkk1-E4. The wild-type *mkk1* allele was amplified using the primer pair Mkk1-E1/Mkk1-E4. In both cases they produced a 2.1 kb fragment flanked by *Nde*I and *Eco*RI sites that were cloned into pRU11 an integrative *U. maydis* vector that contains the *crg1* promoter [63] and the resulting plasmid was linearized and integrated into the *ip* locus. To integrate the *mkk1^DDcrg1^* allele into its native locus, the 2.1 kb *Nde*I-*Eco*RI fragment carrying the *mkk1^DD^* allele was ligated together a 0.68 kb *Eco*RI-*Bam*HI fragment carrying the 3′ region of *mkk1* (generated by PCR using the primer pair Mkk1-8E and Mkk1-9B) into the plasmid pRU11-Hyg [67] digested with *Nde*I-*Eco*RI. The resulting plasmid after linearization with *Eco*RI was integrated at the *mkk1* locus.

To produce the alleles *cdc25^scp1^* and *cdc25*Δ*270^scp1^*, we exchanged in the pCdc25nar1 and pCDC25Δ270nar1 plasmids [46] the *nar1* promoter, which is flanked by *Not*I-*Nde*I sites, with a *Not*I-*Nde*I fragment carrying the *scp1* promoter, a weak *U. maydis* constitutive promoter [47].

### Microscopy

Samples were mounted on microscope slides and visualized in a Nikon Eclipse 90i microscope equipped with a Hamamatsu ORCA-ER CCD camera. All the images in this study are single planes. Standard DAPI, rhodamine and GFP filter sets were used for epifluorescence analysis. The software used with the microscope was MetaMorph 7.1 (Universal Imaging, Downingtown, PA). Images were further processed with Adobe Photoshop 8.0.

To quantify the ratio of the nuclear intensity (N) to the cytoplasmic intensity (C) of Cdc25-GFP signal already described procedures were followed [50]. Briefly, the intensity of the nuclear and cytoplasmic signal was determined by measuring pixel intensity in the nucleus and of an equivalent area in the cytoplasm and the ratio was determined. 30 cells were quantified for each experiment.

## Supporting Information

Figure S1 Dendrogram analysis of MEKK, MEK and MAPK extracted from *U. maydis* database and compared with *S. cerevisiae* and *S. pombe*. Branches were named after the *S. cerevisiae* MAPK pathway.
(0.30 MB TIF)

Figure S2 Levels of Thr193 and Tyr195 Mpk1 phosphorylation after treatment with cell wall stressors. Protein extracts from the indicated strains growing in the presence of cell wall stressors for the indicated time (minutes) were separated by SDS-PAGE. Immunoblots were incubated successively with an antibody that recognizes the Mpk1 phosphorylated form (anti-pTEpY) and anti-HA. The levels of phosphorylation were determined by quantifying the level of antibody signal using a ChemiDoc (Bio-Rad). Signal from the phosphopeptide-specific antibodies was normalized to the amount of phosphorylation of at time zero. Differences in loading of samples were corrected by dividing each phosphopeptide-specific antibody signal by the anti-HA antibody signal. Mean and s.d. are shown (n = 3 experiments).
(0.03 MB PDF)

Figure S3 Expression of *mkk1^DD^* resulted in cell lysis. Strain UMA7 was grown at 28°C in YPA for 8 h. Weakness of cell wall at the tips was apparent by the leakage of cell material.
(0.09 MB PDF)

Figure S4 Deletion of *mpk1* gene abolishes the morphological defects observed *upon* expression of *mkk1^DD^.* Strains FB1 (wt), UMA7 (*P_crg1_: mkk1^DD^*) and UMA13 (*P_crg1_: mkk1^DD^ mpk1*Δ) were grown in YPA at 28°C for 8 h.
(1.14 MB PDF)

Figure S5 Clb2-GFP is a marker of G2 phase in *U. maydis*. A. Scheme of cell cycle in *U. maydis* where different phases are associated to specific morphological markers: microtubules, nuclear membrane, and presence or absence of buds (based in results from different sources: [Perez-Martin, et al; Steinberg, et al; Straube, et al]). Presence of Clb2-GFP fluorescence is also included from our data. Cells without buds are in G1 and S phase. Cells with buds, one single nucleus surrounded by an intact nuclear membrane, and a cytoplasmic array of MT are G2 cells. Cells with large buds, mitotic spindle and nuclear membrane partially disassembled are in mitosis and finally cells with large buds, two nuclei (one in the bud and other in mother cell), cytoplasmic array of microtubules and in the process of cell separation are early G1 cells. B. Correlation between presence of GFP signal (GFP+) and G2 phase. A strain carrying Clb2-GFP and Cut11-RFP fusions was grown in YPD until mid-log phase. 200 cells were counted and sorted in 4 different groups depending on presence or absence of bud, number of nuclei and integrity of nuclear membrane. C. Cell images of cells carrying Clb2-GFP and Cut11-RFP fusion showing different cell cycle phases. [Perez-Martin J, Castillo-Lluva S, Sgarlata C, Flor-Parra I, Mielnichuk N, et al. (2006) Pathocycles: Ustilago maydis as a model to study the relationships between cell cycle and virulence in pathogenic fungi. Mol Genet Genomics 276: 211-229. Steinberg G, Wedlich-Soldner R, Brill M, Schulz I (2001) Microtubules in the fungal pathogen Ustilago maydis are highly dynamic and determine cell polarity. J Cell Sci 114: 609-622. Straube A, Weber I, Steinberg G (2005) A novel mechanism of nuclear envelope break-down in a fungus: nuclear migration strips off the envelope. EMBO J 24: 1674-1685.]
(2.32 MB TIF)

Figure S6 Ectopic expression of *wee1* does not preclude accelerated G2/M transition upon over-activation of Mpk1. (A) DIC and DAPI images of UMA20 (*mkk1^DDcrg1^*), UMC48 (*P_crg1_:wee1*) and UMA79 (*P_crg1_:wee1 mkk1^DDcrg1^*) cells grown for 8 h in YPA at 22°C. Bar 15 µm. In this experiment, we wondered whether high levels of *wee1* expression were able to preclude the G2 shortening observed after over-activation of Mpk1. To address this question we introduced an ectopic copy of a MYC-tagged *wee1* allele under the control of *crg1* promoter in a strain carrying the *mkk1^DDcrg1^* allele. By this manipulation, over-activation of Mpk1 (upon expression of the *mkk1^DDcrg1^* allele) will occur concomitantly with over-expression of *wee1*. We found that high levels of Wee1 in a control strain produced elongated buds with a single nucleus (A, middle column), concomitant with a G2 cell cycle arrest. However, when *wee1* was overexpressed in cells carrying the *mkk1^DDcrg1^* allele, the presence of high levels of Wee1 did not prevent the G2 shortening. The observed cell compartments in *wee1*-overexpressing strain were slightly larger than those observed in the strain not expressing *wee1* (182 µm^3^ in cells overexpressing *wee1* versus 104 µm^3^ in cells not overexpressing *wee1* on average for cell aggregates carrying at least four cell compartments). (B) Western blot showing the levels of Wee1-myc in the above strains, after grown for 8 h under inducing (YPA) and repressing (YPD) conditions for *crg1*. As loading control Cdk1 and Cdk5 levels were analyzed using anti-PSTAIRE antibodies.
(1.15 MB TIF)
