# Supplementary figures and images for: Activation of the Cell Wall Integrity Pathway Promotes Escape from G2 in the Fungus Ustilago maydis
Source: PLoS Genet. 2010 Jul 1;6(7):e1001009. doi: 10.1371/journal.pgen.1001009 (PMC2895642; doi:10.1371/journal.pgen.1001009)

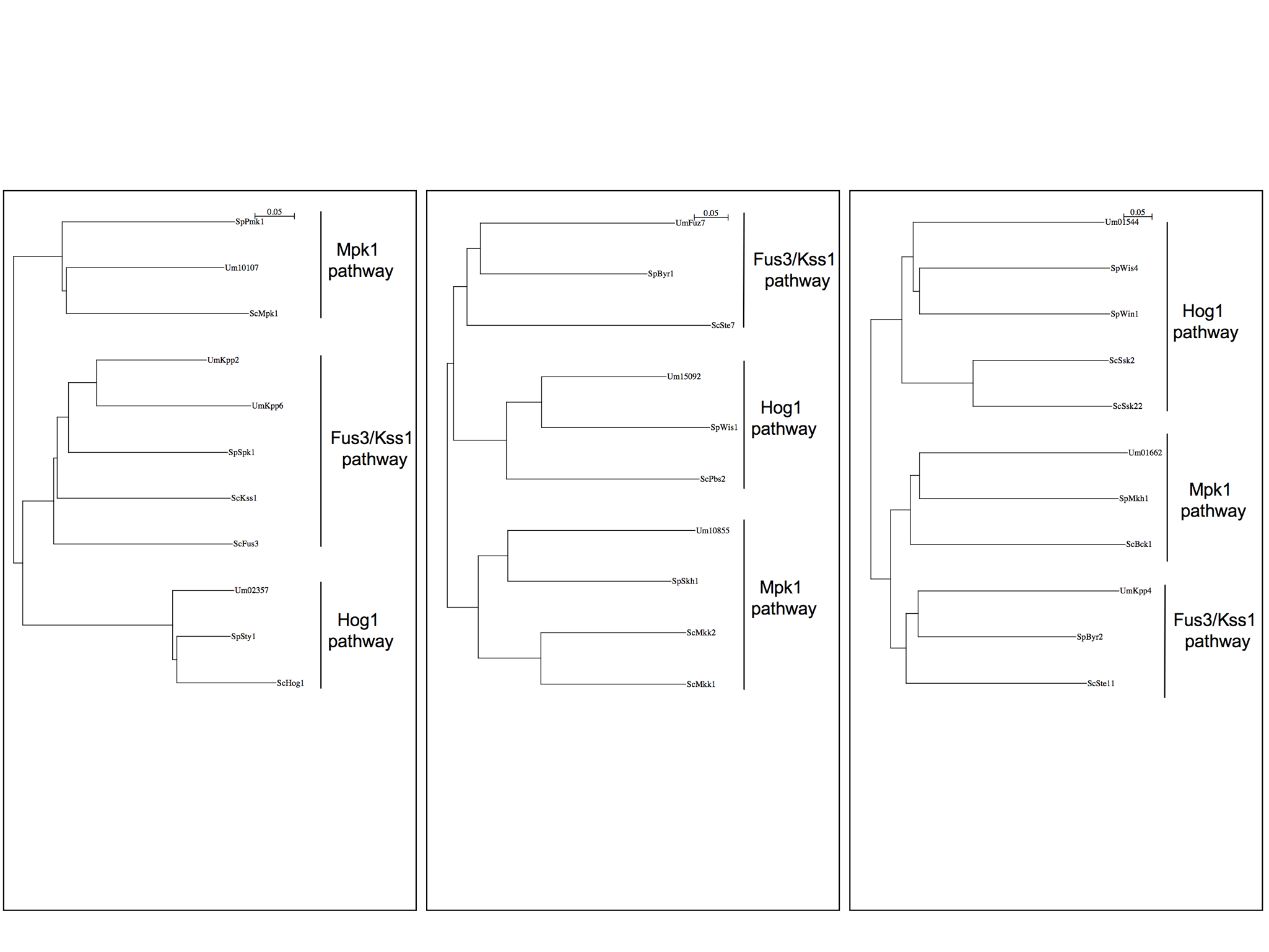

Supplement: Figure S1 — Dendrogram analysis of MEKK, MEK and MAPK extracted from U. maydis database and compared with S. cerevisiae and S. pombe. Branches were named after the S. cerevisiae MAPK pathway. (0.30 MB TIF) [file pgen.1001009.s001.tif]

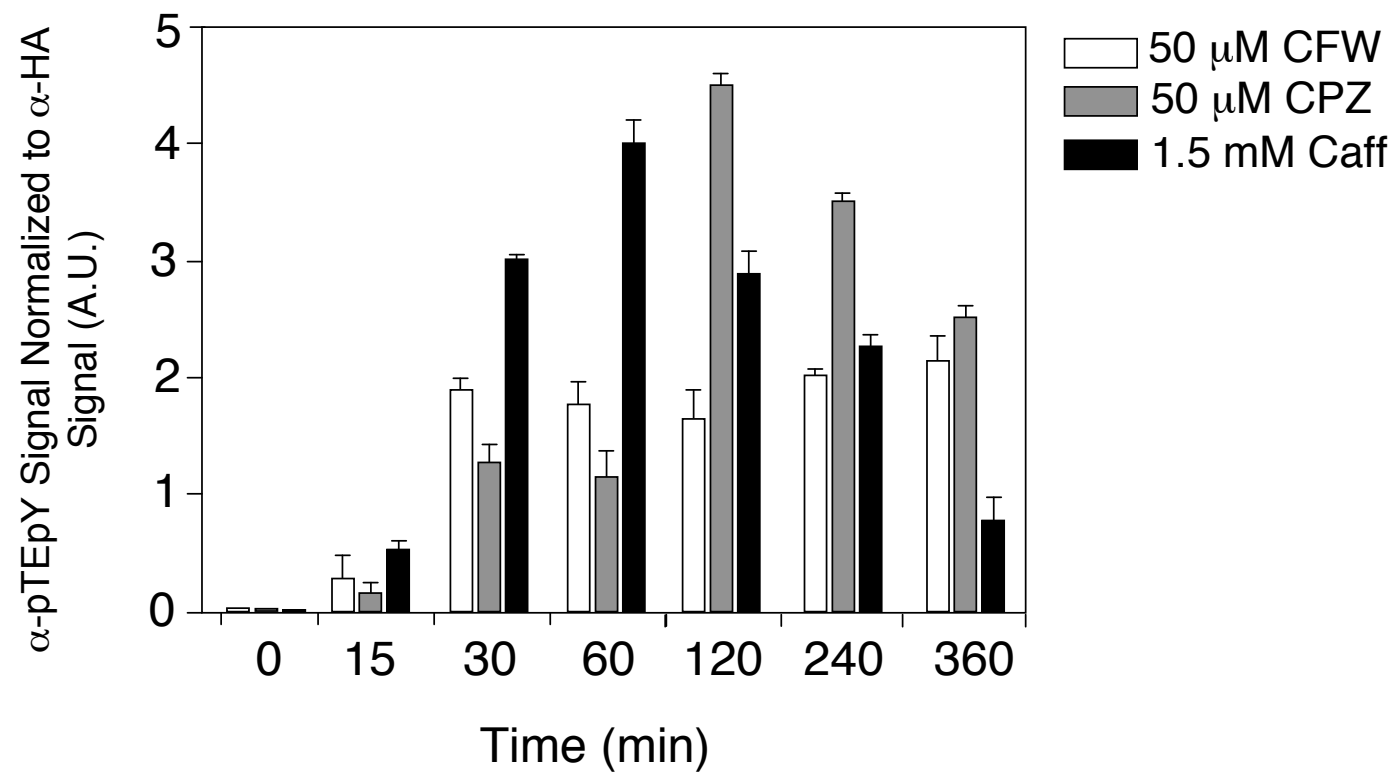

Supplement: Figure S2 — Levels of Thr193 and Tyr195 Mpk1 phosphorylation after treatment with cell wall stressors. Protein extracts from the indicated strains growing in the presence of cell wall stressors for the indicated time (minutes) were separated by SDS-PAGE. Immunoblots were incubated successively with an antibody that recognizes the Mpk1 phosphorylated form (anti-pTEpY) and anti-HA. The levels of phosphorylation were determined by quantifying the level of antibody signal using a ChemiDoc (Bio-Rad). Signal from the phosphopeptide-specific antibodies was normalized to the amount of phosphorylation of at time zero. Differences in loading of samples were corrected by dividing each phosphopeptide-specific antibody signal by the anti-HA antibody signal. Mean and s.d. are shown (n = 3 experiments). (0.03 MB PDF) [file pgen.1001009.s002.pdf]

*P<sub>crg1</sub>:mkk1<sup>DD</sup>*

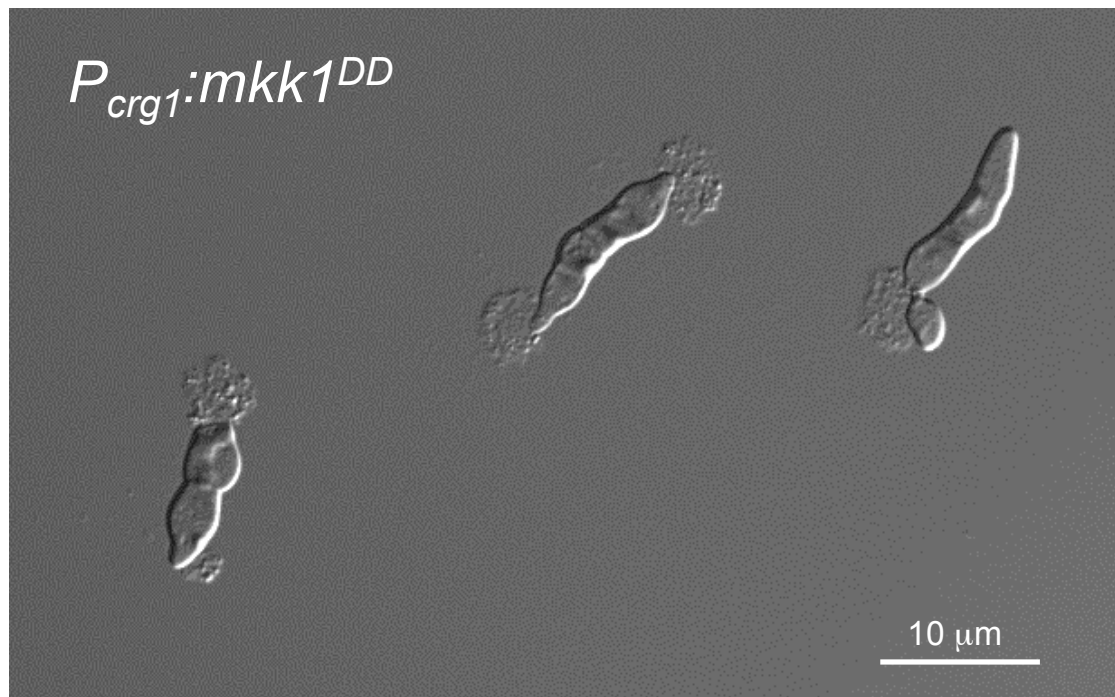

Supplement: Figure S3 — Expression of mkk1DD resulted in cell lysis. Strain UMA7 was grown at 28°C in YPA for 8 h. Weakness of cell wall at the tips was apparent by the leakage of cell material. (0.09 MB PDF) [file pgen.1001009.s003.pdf]

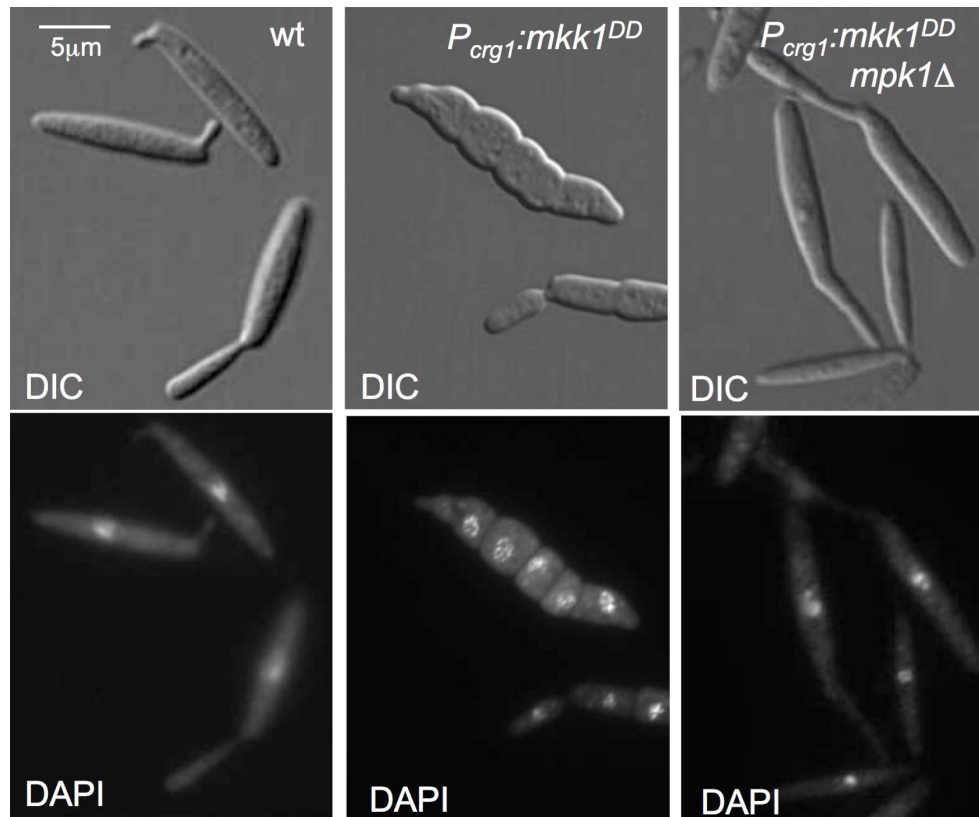

Supplement: Figure S4 — Deletion of mpk1 gene abolishes the morphological defects observed upon expression of mkk1DD. Strains FB1 (wt), UMA7 (Pcrg1: mkk1DD) and UMA13 (Pcrg1: mkk1DD mpk1Δ) were grown in YPA at 28°C for 8 h. (1.14 MB PDF) [file pgen.1001009.s004.pdf]

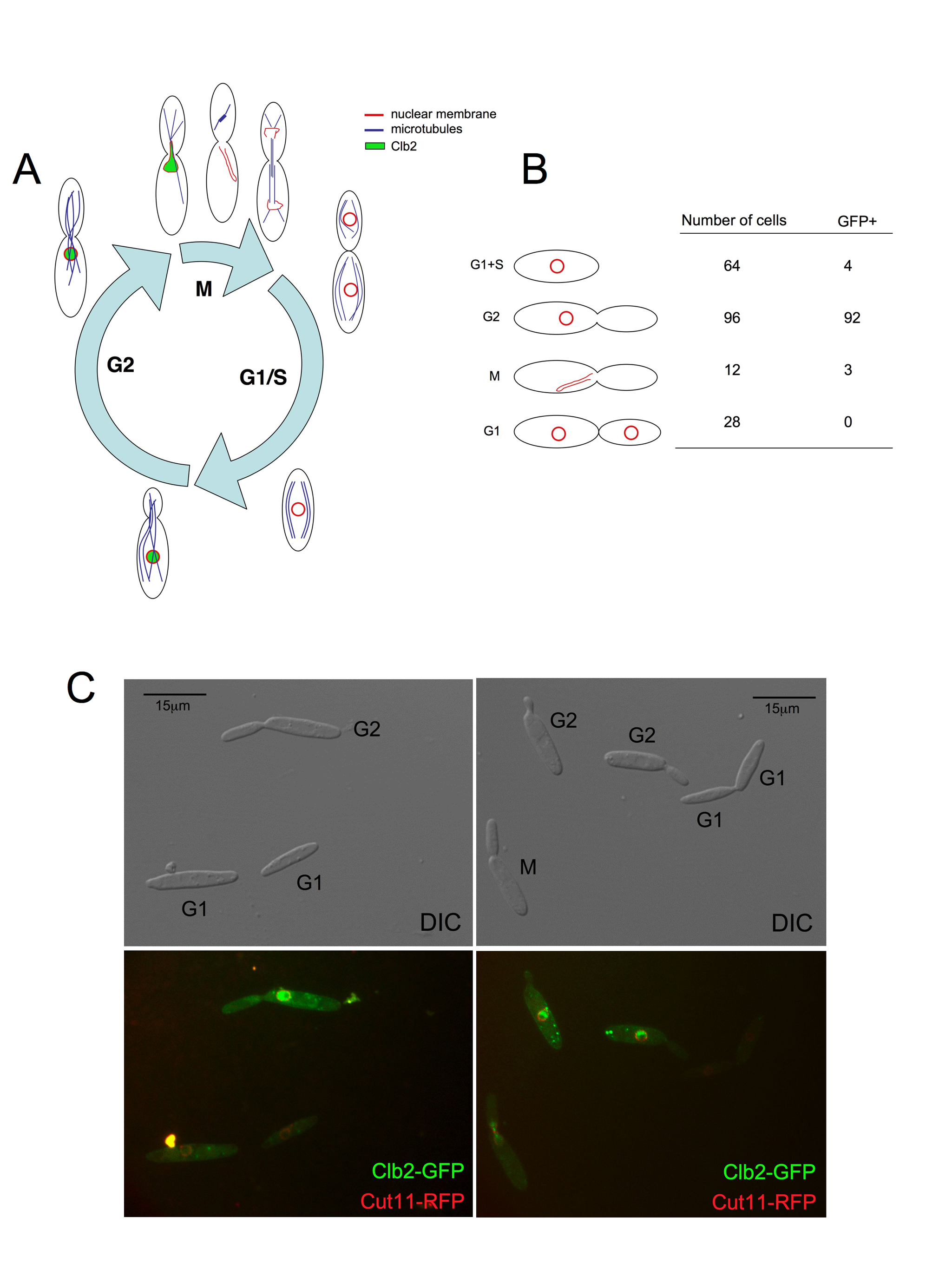

Supplement: Figure S5 — Clb2-GFP is a marker of G2 phase in U. maydis. A. Scheme of cell cycle in U. maydis where different phases are associated to specific morphological markers: microtubules, nuclear membrane, and presence or absence of buds (based in results from different sources: [Perez-Martin, et al; Steinberg, et al; Straube, et al]). Presence of Clb2-GFP fluorescence is also included from our data. Cells without buds are in G1 and S phase. Cells with buds, one single nucleus surrounded by an intact nuclear membrane, and a cytoplasmic array of MT are G2 cells. Cells with large buds, mitotic spindle and nuclear membrane partially disassembled are in mitosis and finally cells with large buds, two nuclei (one in the bud and other in mother cell), cytoplasmic array of microtubules and in the process of cell separation are early G1 cells. B. Correlation between presence of GFP signal (GFP+) and G2 phase. A strain carrying Clb2-GFP and Cut11-RFP fusions was grown in YPD until mid-log phase. 200 cells were counted and sorted in 4 different groups depending on presence or absence of bud, number of nuclei and integrity of nuclear membrane. C. Cell images of cells carrying Clb2-GFP and Cut11-RFP fusion showing different cell cycle phases. [Perez-Martin J, Castillo-Lluva S, Sgarlata C, Flor-Parra I, Mielnichuk N, et al. (2006) Pathocycles: Ustilago maydis as a model to study the relationships between cell cycle and virulence in pathogenic fungi. Mol Genet Genomics 276: 211-229. Steinberg G, Wedlich-Soldner R, Brill M, Schulz I (2001) Microtubules in the fungal pathogen Ustilago maydis are highly dynamic and determine cell polarity. J Cell Sci 114: 609-622. Straube A, Weber I, Steinberg G (2005) A novel mechanism of nuclear envelope break-down in a fungus: nuclear migration strips off the envelope. EMBO J 24: 1674-1685.] (2.32 MB TIF) [file pgen.1001009.s005.tif]

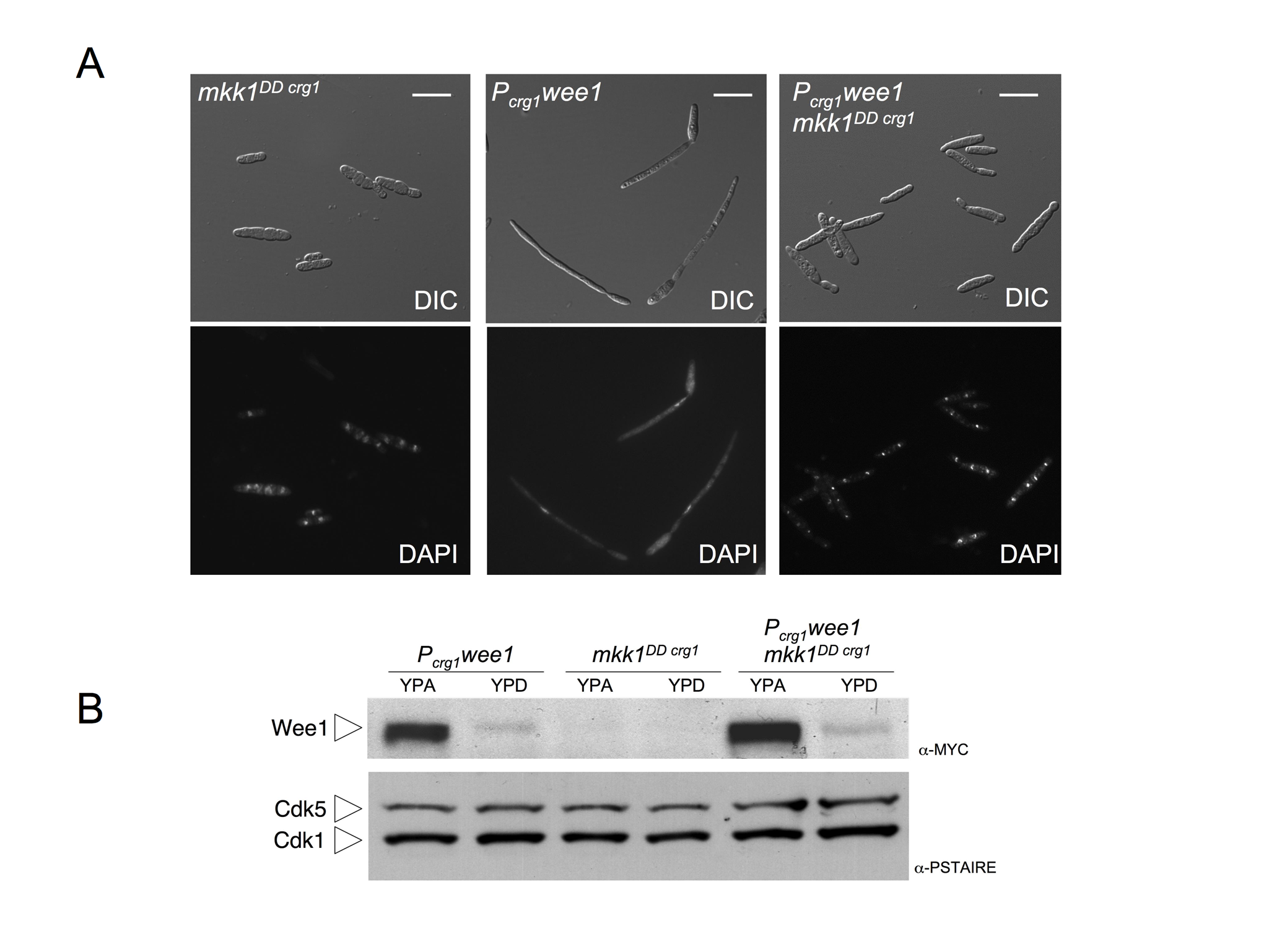

Supplement: Figure S6 — Ectopic expression of wee1 does not preclude accelerated G2/M transition upon over-activation of Mpk1. (A) DIC and DAPI images of UMA20 (mkk1DDcrg1), UMC48 (Pcrg1:wee1) and UMA79 (Pcrg1:wee1 mkk1DDcrg1) cells grown for 8 h in YPA at 22°C. Bar 15 µm. In this experiment, we wondered whether high levels of wee1 expression were able to preclude the G2 shortening observed after over-activation of Mpk1. To address this question we introduced an ectopic copy of a MYC-tagged wee1 allele under the control of crg1 promoter in a strain carrying the mkk1DDcrg1 allele. By this manipulation, over-activation of Mpk1 (upon expression of the mkk1DDcrg1 allele) will occur concomitantly with over-expression of wee1. We found that high levels of Wee1 in a control strain produced elongated buds with a single nucleus (A, middle column), concomitant with a G2 cell cycle arrest. However, when wee1 was overexpressed in cells carrying the mkk1DDcrg1 allele, the presence of high levels of Wee1 did not prevent the G2 shortening. The observed cell compartments in wee1-overexpressing strain were slightly larger than those observed in the strain not expressing wee1 (182 µm3 in cells overexpressing wee1 versus 104 µm3 in cells not overexpressing wee1 on average for cell aggregates carrying at least four cell compartments). (B) Western blot showing the levels of Wee1-myc in the above strains, after grown for 8 h under inducing (YPA) and repressing (YPD) conditions for crg1. As loading control Cdk1 and Cdk5 levels were analyzed using anti-PSTAIRE antibodies. (1.15 MB TIF) [file pgen.1001009.s006.tif]
